# Bile acids-gut microbiota crosstalk contributes to the improvement of type 2 diabetes mellitus

**DOI:** 10.3389/fphar.2022.1027212

**Published:** 2022-10-25

**Authors:** Ruolin Gao, Xiangjing Meng, Yili Xue, Min Mao, Yaru Liu, Xuewen Tian, Bo Sui, Xun Li, Pengyi Zhang

**Affiliations:** ^1^ School of Sports and Health, Shandong Sport University, Jinan, China; ^2^ Shandong Academy of Pharmaceutical Science, Jinan, China; ^3^ School of Nursing and Rehabilitation, Shandong University, Jinan, China

**Keywords:** bile acids, gut microbiota, type 2 diabetes mellitus, insulin sensitivity, enterohepatic cycling

## Abstract

Type 2 diabetes mellitus (T2DM) occurs that cannot effectively use the insulin. Insulin Resistance (IR) is a significant characteristic of T2DM which is also an essential treatment target in blood glucose regulation to prevent T2DM and its complications. Bile acids (BAs) are one group of bioactive metabolites synthesized from cholesterol in liver. BAs play an important role in mutualistic symbiosis between host and gut microbiota. It is shown that T2DM is associated with altered bile acid metabolism which can be regulated by gut microbiota. Simultaneously, BAs also reshape gut microbiota and improve IR and T2DM in the bidirectional communications of the gut-liver axis. This article reviewed the findings on the interaction between BAs and gut microbiota in improving T2DM, which focused on gut microbiota and its debinding function and BAs regulated gut microbiota through FXR/TGR5. Meanwhile, BAs and their derivatives that are effective for improving T2DM and other treatments based on bile acid metabolism were also summarized. This review highlighted that BAs play a critical role in the glucose metabolism and may serve as therapeutic targets in T2DM, providing a reference for discovering and screening novel therapeutic drugs.

## 1 Introduction

A total of 537 million people live with diabetes globally according to the International Diabetes Federation in 2021. Among them, there are 140.9 million adults (26.2%) were in China, ranking first in the world. In 2021, the prevalence of diabetes in China was 10.6%, slightly lower than in the United States (10.7%) (International Diabetes Federation, 2021). The high prevalence of diabetes in China is, on one hand, due to the increasing aging population; and on the other hand, is related to the lack of physical activities and the refined grain-based diet. Type 2 diabetes mellitus (T2DM), accounting for 90%–95% of all diabetes, is one of the most common chronic metabolic diseases in the world. The main pathophysiological pathogenesis of T2DM is due to Insulin Resistance (IR) and insufficient insulin production of pancreatic *ß* cells (Cook et al., 2015). Recent research focuses on the human gut microbiota on the intervention of T2DM development. A high-fat, high-sugar diet often leads to a decline in the health of the gut microbiota, which leads to the ecological imbalance in human guts. Gut microbiota imbalance can lead to metabolic dysregulation, including increased IR and elevated levels of inflammation. These are the two key factors in the development of T2DM. Bile acids (BAs) homeostasis is found to be disrupted in T2DM patients, suggesting that BAs interacts with the gut microbiota to regulate carbohydrate, lipid and energy metabolism (Zhang et al., 2018). Meanwhile, BAs regulates the composition of gut microbiota by activating TGR5/FXR receptors, which maintains the stability of intestinal environment, and in turns, improves T2DM (Cipriani et al., 2011; Kakiyama et al., 2013). Furthermore, enzymes synthesized by gut microbiota alter the composition pattern of secondary bile acids (SBAs), which affects the regulation process of carbohydrate and lipid metabolism mediated by various SBAs. This contributes to the improvement of T2DM (Wahlström et al., 2016). Therefore, the dietary intervention of gut microbiota regulating the secretion and metabolism of BAs may help prevent and inhibit inflammatory metabolic disorders, such as T2DM.

Cholesterol is oxidized by enzymes in the liver and becomes the material to produce many different BAs metabolized by gut microbiota. The role of BAs is to facilitate digestion and absorption of fats from food, as well as to activate hormones through different receptors. BAs are the main organic components of bile, which promote the absorption of dietary lipids and maintain cholesterol homeostasis (Jia and Yang, 2019). In addition, BAs act as the significant signaling molecules in the regulation of energy homeostasis to activate different receptors, which play an important role in pathogenesis of T2DM (Perino and Schoonjans, 2022). Recent studies showed that bile acid metabolism altered in T2DM patients, suggesting T2DM was associated with composition and proportion changes of BAs (Sonne et al., 2016; Pandak and Kakiyama, 2019). Alter the bile acid composition may improve blood glucose regulation in T2DM patients. BAs as metabolic regulators participate in the regulation of glucose and lipid metabolism by activating different signaling pathways. Therefore, this article reviewed the findings on the signaling transduction of BAs and their receptors in the improvement of T2DM, the crosstalk with gut microbiota, and their potential therapeutic applications.

## 2 Synthesis and metabolism of bile acids

BAs are synthesized by cholesterol in the liver and its physiological functions are to participate in the absorption of nutrients and fat-soluble vitamins in the intestine, promote the metabolism of lipids and toxic metabolites, and regulate the glucose metabolism through various mechanisms (Meier and Stieger, 2002). The conversion of cholesterol to BAs is critical for maintaining the cholesterol circulation and preventing the accumulation of cholesterol and triglycerides that cause damage to the liver and other organs. BAs are also signaling molecules and metabolic regulators, which can activate nuclear receptors and G protein coupled receptors (GPCRs) signals, regulate liver lipid and glucose metabolism, and maintain energy homeostasis (Kumar et al., 2016). The enterohepatic cycling of BAs between liver and intestine plays a critical role in nutritional absorption and metabolic regulation. This physiological process is regulated by a complex membrane transport system *via* nuclear receptors. The disorder of bile acid metabolism can lead to cholestatic liver disease, fatty liver, dyslipidemia, cardiovascular disease, and diabetes (Jia et al., 2021). Several studies have shown the potential value of BAs, BA derivatives and BA sequestrants for the treatment of metabolic liver disease, obesity, and diabetes (Potthoff et al., 2013; Kumar et al., 2016; Guo et al., 2021; Mooranian et al., 2022).

BA synthesis is the main pathway for cholesterol catabolism. The BA production pathways mainly include the classical (or neutral) pathway and the alternative (or acidic) pathway. Cholesterol initiates the neutral (or classical) pathway to synthesize BAs in hepatocytes *via* cholesterol 7α-hydroxylase (CYP7A1) located on the smooth endoplasmic reticulum. The elevated hepatic cholesterol level induces the formation of oxysterols, which bind to liver X receptor *α* (LXRα) and activate CYP7A1 transcription. Cholesterol is catalyzed by CYP7A1 to produce 7α-hydroxycholesterol, which is further catalyzed to produce chenodeoxycholic acid (CDCA) and cholic acid (CA). The upregulation of CYP7A1 expression can increase BA synthesis and cholesterol excretion. Subsequently, BAs act as the signaling molecules to activate farnesoid X receptor (FXR) and Takeda G-protein receptor 5 (TGR5) to regulate body metabolism. CYP7A1 is the rate-limiting enzyme of the entire pathway and determines the amounts of BAs. At least 75% of BAs are produced through this pathway under common conditions. In the alternative (or acidic) pathway cholesterol is catalyzed by sterol-27-hydroxylase (CYP27A1) to produce 27-hydroxycholesterol, which is further catalyzed by oxysterol-7α-hydroxylase (CYP7B1) to produce CDCA. Humans and mice produced 9% and 25% of total BAs by alternative pathways, respectively (Wahlström et al., 2016).

**FIGURE 1:**
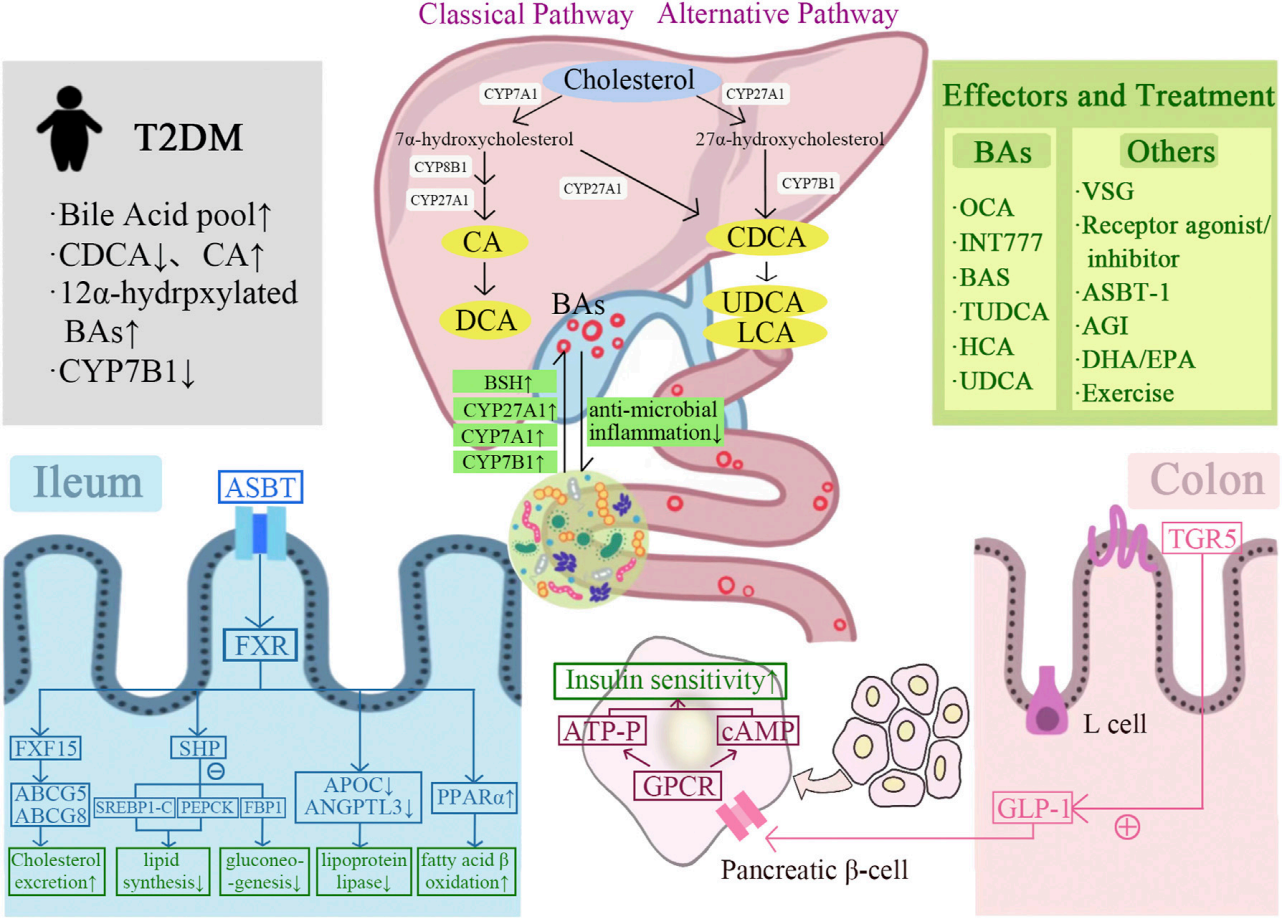
Graphical representation of the mechanism of bile acids-gut microbiota crosstalk improves T2DM. BAs are synthesized from cholesterol in the liver *via* classical and alternative pathways and interact with the gut microbiota. BAs improve insulin sensitivity and T2DM by regulating FXR/TGR5, glucose and lipid metabolism, and GLP-1 secretion. A variety of BAs and their derivatives contribute to the treatment of T2DM, including OCA, INT77, BAS, TUDCA, HCA, and UDCA. In addition, T2DM can also be improved by other treatments, including bariatric surgery, receptor agonists/inhibitors, ASBT-1, AGI, DHA/EPA, and exercise.

BAs synthesized in liver are secreted *via* the bile, which are mainly reabsorbed in intestine and transported back to liver. After a meal, cholecystokinin secreted by the intestine stimulates the contraction of the gallbladder, and BAs stored in the gallbladder enter the gut. In the enterohepatic cycling, most of BAs (95%) are reabsorbed in the terminal ileum *via* the apical sodium-dependent bile acid transporter (ASBT) (Jia et al., 2021). The reabsorbed BAs diffuse through the enterocytes to the outside of the basement membrane and circulates through the portal vein to the hepatic sinuses, finally are absorbed by the hepatocytes. Some BAs are reabsorbed in biliary epithelial cells and circulate back to hepatocytes. Small amounts of BAs may leak into the systemic circulation, are reabsorbed as they pass through renal tubules of kidneys, and then return to liver *via* the systemic circulation (Li and Chiang, 2014). The total amount of BAs and bile salts in the enterohepatic cycling is called the bile acid pool. The bile acid composition in humans is different from that in mice. In humans, the highly hydrophobic bile acid pool consists of approximately 40% CA, CDCA, and 20% DCA. In mice, the highly hydrophilic bile acid pool consists of approximately 50% CA, 50% Tauro-α muricholic acid and Tauro-β muricholic acid (T-αMCA and T-βMCA) (Li and Chiang, 2014). BAs play an important role in glucose and lipid metabolism. Therefore, it may be an effective way to treat T2DM by regulating the composition of BAs *via* bile acid synthase. The major products of BAs are CDCA and CA. Physiologically, CDCA can dissolve gallstones and ensure smooth enterohepatic cycling of BAs, and CA can promote the digestion and absorption of lipids. CDCA and CA not reabsorbed by ASBT enter the colon, where they bind to glycine or taurine and are stored in the gallbladder. After feeding stimulation, CDCA and CA enter the intestine and are uncoupled and dihydroxylated by gut microbiota to produce SBAs such as ursodeoxycholic acid (UDCA) and lithocholic acid (LCA) (Figure 1) (Wahlström et al., 2016).

## 3 Bile acids and gut microbiota

The gut microbiota plays an important role in the regulation of bile acid metabolism. The composition of SBAs can be altered by the enzymes synthesized by gut microbiota, and then affect the regulation of glucose and lipid metabolism mediated by various SBAs (Zhang et al., 2018). The BAs also interact with the gut microbiota, and the composition difference of BAs can explain 37% of the distribution difference of gut microbiota (Chiang and Ferrell, 2019). The physiological functions of BAs are mediated by gut microbiota. The gut microbiota uncouples conjugated BAs to free BAs *via* bile salt hydrolases, and then dehydrogenates or dehydroxylates the free BAs to SBAs, such as converting CA to deoxycholic acid (DCA) and CDCA to LCA. DCA is reabsorbed in the colon and circulates to the liver with CA and CDCA. A bile acid pool of about 3 g, consisting of about 40% CA, 40% CDCA, 20% DCA, and a trace amount of LCA, was recovered 4–12 times per day. The remaining 5% of BAs that do not enter the enterohepatic cycling, part of which is catabolized by gut microbiota to generate SBAs, which are passively reabsorbed by the small intestine and colon; the other part is excreted through feces (Chiang and Ferrell, 2019; Ticho et al., 2019). The gut microbiota involved in converting primary BAs into SBAs are *Bacteroides* *, Clostridium, Eubacterium, Lactobacillus and Escherichia* (Ticho et al., 2019).

### 3.1 Gut microbiota and their metabolites

The basic functions of gut microbiota are to participate in the regulation of intestinal metabolites. It is estimated that more than 8,000 non-nutritive compounds exist in the human gut, most of which cannot be digested by human digestive enzymes (Wishart, 2019). The carbohydrates, lipids, proteins, and complex dietary fibers are fermented and catabolized by gut microbiota in the colon to produce functional metabolites, such as short-chain fatty acids (SCFAs), SBAs, and hormones. The colon is the primary site of fermentation, but gut microbiota from other sites, such as *Lactobacillus* and *Streptococcus* in small intestine, also participate in the regulation of nutrient metabolism (Liang et al., 2021). The dysbiosis of gut microbiota leads to changes of many metabolites, of which the type and concentration affect the host-sensing receptor responses (Chen et al., 2019).

### 3.2 Gut microbiota regulates bile acids

#### 3.2.1 Deconjugation of gut microbiota regulates the composition of bile acid pool

The deconjugation of BAs by gut microbiota is carried out in the small intestine by bacteria with bile salt hydrolase (BSH) activity, thus maintaining normal levels of deconjugated bile acids and cholesterol in the circulation (Chiang, 2013). Metagenomics studies have shown that all major bacteria and archaea in the human intestine express functional BSH, including *Lactobacillus, Bifidobacterium, Clostridium, Bacteroides*, *etc.* (Odermatt et al., 2011). BSH enriched in gut microbiota is related to the increased resistance to bile toxicity. The antibiotic use and the dysbiosis of gut microbiota are associated with long-term changes in gut microbiota composition and bile acid metabolism. BSH activity is necessary to accelerate cholesterol catabolism by increasing the excretion of bile acids (Supplementary Table S1) (Chiang, 2013). The study showed that mice treated with 3 days of oral antibiotics exhibit impaired bile acid metabolism similar to germ-free animals (Miyata et al., 2009). In summary, gut microbiota and its deconjugation function led to changes in the composition of bile acid pool that contribute to the maintenance of cholesterol homeostasis.

#### 3.2.2 Gut microbiota regulates the expression of bile acid synthase

The gut microbiota regulates bile acid production by regulating the expression of the rate-limiting enzymes cholesterol-7α-hydroxylase (CYP7A1), CYP27A1, and CYP7B1 in the bile acid production pathway (Wahlström et al., 2016). Excessive amount of cholesterol is removed from the peripheral system, including lipid-filled macrophages, *via* the reverse cholesterol transport (RCT) pathway. During this process, cholesterol is converted to BA by CYP7A1, which is subsequently excreted in the feces (Chambers et al., 2019). By crossing Cyp7A1 and Cyp27A1 gene deficient mice, the total amount of BA in the double-gene knockout mice was reduced more than 85%, demonstrating that the deletion of the rate-limiting enzyme in the BA production pathway caused the decrease of BA synthesis (Rizzolo et al., 2021). It was shown that the number of 7-dehydroxylase-producing bacteria was significantly reduced in the intestine of germ-free or antibiotic-treated mice, and the levels of glycine-conjugated BAs and SBAs were also significantly reduced, while the levels of taurine conjugated bile acid (TBA) were significantly increased (Wahlström et al., 2016). In addition, the antibiotic treatment can upregulate the expression of CYP7A1 and increase the production of CDCA and CA by inhibiting the expression of fibroblast growth factor 15 (FGF15) (Sayin et al., 2013; Chambers et al., 2019). All the above findings indicated gut microbiota played a regulatory role in BAs synthesis (Figure 1).

#### 3.2.3 Probiotics regulates bile acid synthesis

Probiotics are involved in the regulation of bile acid synthesis and metabolism during the interaction of bile acids and gut microbiota. The currently known mechanisms include: 1) regulation of BSH activity *Bifidobacterium* and *Lactobacillus* were identified as probiotics with BSH activity, among which *Bifidobacterium breve* and *Lactobacillus plantarum LA3* strains had the highest BSH activity (Öner et al., 2014). BSH catalyzes the conversion of primary BAs to SBAs, leading to reduced reabsorption of BAs in the enterohepatic circulation and easier excretion of SBAs from the gut, thereby promoting bile acid synthesis and LDL-cholesterol clearance (Costabile et al., 2017; Pushpass et al., 2021). 2) assimilation of cholesterol Probiotics lower cholesterol levels by binding cholesterol to their cell membranes and converting it into coprostanol (Lye et al., 2010). 3) activation/inhibition of FXR DegGirolamo et al. found that the bile acid composition was significantly altered in mice after gavage with the probiotic mixture VSL#3 for 21 days. The high levels of BSH activity inhibited FXR response in the gut-liver axis and resulted in a decrease in the ratio of conjugated BAs/free BAs in feces (Degirolamo et al., 2014). In contrast, Choi et al. found that the addition of *Lactobacillus. Curvatus* and *Lactobacillus. Plantarum* to a high-fat diet for 6 weeks increased the intestinal FXR expression in mice (Inagaki et al., 2005). Martoni et al. found that supplementation with *Lactobacillus reuteri* activated FXR as well as increased circulating bile acid levels (Martoni et al., 2015).

*Akkermansia muciniphila (Akk)* accounts for 1%–5% of the human gut microbiota and is considered to be a new generation of beneficial microorganisms (Zhang et al., 2019). *Akk* treatment has been verified to improve symptoms of metabolic disorders, including IR, metabolic endotoxemia, and adipose tissue inflammation (Zhao et al., 2017; Depommier et al., 2019). Several studies revealed how *Akk* regulated signal transduction and energy expenditure to achieve its metabolic effect mechanisms (Grander et al., 2018; Sheng et al., 2018). *Akk* supplementation reduced the synthesis and transport of fatty acid in the liver and muscle, and attenuated chronic low-grade inflammation by inhibiting the lipopolysaccharide (LPS)-binding protein (LBP) signaling pathway (Zhao et al., 2017). Another study found that *Akk* treatment increased hepatic absorption of cholesterol and bile acid metabolism, and improved the enterohepatic cycling of bile acid metabolism. In turn, the enhanced bile acid metabolism facilitated the remodeling of gut microbiota composition and the colonization of *Akk* (Rao et al., 2021). In addition, the activation of oxidative stress and the induction of intestinal cell apoptosis damage the intestinal barrier, leading to the development of diseases such as inflammatory bowel disease (IBD), obesity and T2DM (Aviello et al., 2019; Franzosa et al., 2019; Ni et al., 2021). *Akk* treatment effectively increased lipid oxidation, ameliorated lipid accumulation-induced intestinal oxidative stress and cell apoptosis, and maintained them at normal levels (Rao et al., 2021). These findings further confirm the potential of *Akk* in the treatment of metabolic disorders.

Clearly, we need to provide more details through human experiments to elucidate the mechanisms behind the beneficial effects of specific probiotic-bile acid interactions on glycolipid metabolism.

### 3.3 Bile acids regulate gut microbiota via farnesoid X receptor/Takeda G-protein receptor 5

BAs is an important regulator of gut microbiota balance. Low bile acid levels appeared to reduce the number of Gram-positive bacteria. Reduced bile acid levels in the intestine appear to favor Gram-negative bacteria in the gut microbiota, some of which are potentially pathogenic bacteria that produce strong antigenic lipopolysaccharides. In contrast, elevated bile acid levels in the intestine favored the transformation of Gram-positive bacteria *Firmicutes*, including bacteria that caused dehydroxylation of primary BAs to harmful SBAs. The complex and significant changes in gut microbiota at the phylum level were observed in rats fed CA-containing diet. Compared with the control group, the proportion of *Firmicutes* expanded from 54% to 93%–98%, and *Clostridium* expanded from 39% to about 70% (Islam et al., 2011). The SBAs influenced by gut microbiota activate FXR and TGR5 more strongly than the BAs (Long et al., 2017). The BAs have a direct antibacterial effect on the gut microbiota. The SBAs, DCA, is more effective as an antibacterial agent than CA due to its stronger hydrophobicity and detergency on bacterial membranes (Chiang, 2013). Meanwhile, BAs also regulate host immune response through FXR-induced antibacterial peptides or by acting on FXR/TGR5. TGR5 has been shown to be involved in regulating the stability of the intestinal environment. The deletion of TGR5 is manifested by increased intestinal permeability and susceptibility to intestinal inflammation. The overproduction of pro-inflammatory cytokines in FXR^−/−^ mice caused intestinal inflammation. The activating of FXR can prevent the development of inflammation in mice with colitis (Figure 1) (Cipriani et al., 2011).

## 4 Bile acids and type 2 diabetes mellitus

### 4.1 The alterations of bile acid metabolism in type 2 diabetes mellitus

The physiopathology of T2DM is characterized by fasting hyperglycaemia, IR, and postprandial hyperglycemia (Grandl and Wolfrum, 2018). Early and recent studies suggested the size and/or composition of bile acid pool changed in patients with T2DM. The size of the bile acid pools in insulin-treated and untreated T2DM patients were different. The content of CDCA is decreased and the synthesis of CA is increased in bile acid pool in T2DM patients. In human studies, the magnitude of change in postprandial plasma BA levels was positively correlated with dietary fat content. Furthermore, compared to normoglycemic subjects matched with age, sex, and BMI, the T2DM patients had increased total plasma BAs, mainly due to the increased glycine-binding BAs, DCA, and UDCA during oral glucose and dietary tolerance experiments (Sonne et al., 2016). The oral glucose tolerance test was performed on 2952 T2DM patients with normal fasting serum total bile acids (S-TBAs). Results showed that the elevated fasting S-TBAs levels within the normal range were significantly associated with reduced fasting and systemic insulin sensitivity, impaired islet β-cell function and increased blood glucose after glucose stimulation in T2DM patients, suggesting that the regulation of fasting S-TBAs may be a therapeutic target for T2DM (Wang et al., 2020). The plasma concentration of 12α-hydroxylated bile acid was higher in obese and T2DM patients than that in healthy individuals. The transcription level of CYP7B1 was decreased in mice model of IR and T2DM, as well as in obese and T2DM patients (Figure 1) (Chen et al., 2016; Pandak and Chiang, 2019). The studies clearly supported the opinion of altered bile acid metabolism in patients with T2DM.

### 4.2 Bile acids improve insulin sensitivity through receptor-mediated regulation of glucose and lipid metabolism

The BAs can activate a variety of nuclear and membrane receptors. The nuclear receptors mainly include FXR, pregnane X receptor (PXR), steroid and xenobiotic receptor (SXR), constitutional androstane receptor (CAR) and VDR. The membrane receptors mainly include TGR5, sphingosine-1 phosphate receptor 2 (S1PR2) (Ticho et al., 2019). FXR and PXR are abundantly expressed in the liver and intestine. Moreover, they regulate insulin synthesis and glucose-induced secretion in the pancreatic β-cell. VDR is widely distributed in most tissues including pancreas, skin, intestine and liver (Kumar et al., 2016).

#### 4.2.1 Intermediation of nuclear receptor

FXR is the major nuclear receptor for BAs to exert the physiological effect. As the first natural endogenous receptor for BAs, its role in the homeostasis of glucose and lipid metabolism *in vivo* has been confirmed in several studies (Noel et al., 2016). FXR activation improves lipid metabolism and insulin sensitivity in Zucke (fa/fa) obese rats, ob/ob mice, and db/db diabetic mice (Cariou et al., 2006; Cipriani et al., 2010), suggesting that FXR agonists could be used in the treatment of diet-induced T2DM as a pharmaceutical strategy. FXR activation can reduce hepatic lipoprotein synthesis by inducing the gene expression of lipoprotein metabolism or scavenging. At the same time, the expression of triglyceride synthesis genes was inhibited to reduce plasma triglyceride and cholesterol levels (Shapiro et al., 2018). The BAs induce the expression of the target gene SHP by activating hepatic FXR. The SHP reduced hepatic lipid synthesis by inhibiting the expression of the transcription factor sterol regulatory element binding transcription factor-1c (SREBP-1c) and its downstream hepatic lipid synthesis genes (Ahmad and Haeusler, 2019). In addition, the expression of SHP can also reduce hepatic gluconeogenesis by inhibiting phosphoenolpyruvate carboxykinase and fructose bisphosphatase-1 (Mathur et al., 2021). Compared to wild-type mice, the fasting glucose tolerance was significantly impaired in FXR knockout (FXR KO) mice. And the intraperitoneal glucose tolerance and blood glucose fluctuations were significantly improved by injecting the FXR agonist GW4064 in ob/ob and db/db mice (Supplementary Table S1) (Han et al., 2021). The FXR agonist Fexaramine significantly improved the blood glucose levels and reduced the diet-induced weight gain (Fang et al., 2015; Pathak et al., 2018). Furthermore, FXR activation can lead to decreased expression of apolipoprotein C (APOC) and angiopoietin-like protein 3 (ANGPTL3), thereby inhibiting the activity of lipoprotein lipase. In addition, FXR activation induces the expression of peroxisome proliferator-activated receptor *α* (PPARα), which promotes fatty acid β-oxidation (Li and Li, 2017). FXR was activated by BAs to induce the production of FGF15, which changed the ratio of primary BAs to SBAs, enhanced the hydrophilicity of bile, and then allowed cholesterol to be discharged into the intestinal lumen by ATP-binding cassette transport G5 (ABCG5) and ATP-binding cassette transport G8 (ABCG8), resulting in more than 60% of the absorbed cholesterol to be excreted through the intestinal lumen (de Boer et al., 2017).

Several studies have found that the inhibition of FXR also has a significant effect on body glucose and lipid metabolism. Whole-body FXR KO mice and mice lacking FXR in the intestine showed significantly improved oral glucose tolerance and reduced body weight (Chávez-Talavera et al., 2017; Jiang et al., 2015; Trabelsi et al., 2015; Xie et al., 2017). The mechanisms by which FXR inhibition improves glucose and lipid metabolism include: 1) The intestinal FXR-mediated serum ceramide production affects hepatic phosphoenolpyruvate carboxykinase (PEPCK) activity and hepatic gluconeogenesis, and regulates glucose-6-phosphatase (G6P) expression; 2) FXR-dependent inhibition of glucagon-like peptide 1 (GLP-1) precursor increases, thereby promoting GLP-1 production. GLP-1 enhances the glucose-induced insulin secretion and increases the insulin sensitivity in skeletal muscle, adipose tissue, and liver; 3) FXR-dependent inhibition of hepatic glycolytic gene expression increases; 4) Increase the level of glucose phosphorylation in intestinal epithelial cells and delay intestinal glucose absorption (Figure 1). In conclusion, the FXR-mediated regulatory mechanism of glucose and lipid metabolism deserves further in-depth study.

In addition to FXR, VDR can also be activated by LCA and participate in the regulation of glucose and lipid metabolism by affecting pancreatic islets, macrophages, or endothelial cells (Oh et al., 2015; Ni et al., 2016). However, these BAs are difficult to be absorbed into cells. Compared with the activated type of vitamin D, these BAs have a weaker ability to bind the nuclear receptor VDR. Therefore, higher levels of LCA are required to activate the VDR in the body and most occurs in the presence of vitamin D deficiency. At present, the mechanism of BAs-VDR signaling on the homeostasis of glucose and lipid metabolism still needs to be further studied.

#### 4.2.2 Intermediation of membrane receptor

TGR5 is the most intensively studied membrane-bound G protein-coupled receptor, which is expressed in tissues of various metabolic organs, including the intestine, liver, adipose tissue, and muscle. BAs is the only known endogenous ligand for TGR5 (Xiao et al., 2020). The roles of TGR5 in improving glucose and lipid metabolism mainly include: 1) promote hepatic glycogen synthesis and insulin sensitivity; 2) increase insulin secretion from the pancreas; 3) promote energy expenditure, especially in liver, brown adipose tissue, and muscle; 4) facilitate thermogenesis, leading to weight loss; 5) regulate satiety in the brain (Ðanić et al., 2018). After BAs binding, TGR5 activation is related to intracellular accumulation of cyclic adenosine monophosphate (cAMP), adenylate cyclase activation, and calcium mobilization (Duboc et al., 2014). Due to its widespread tissue distribution, BA-activated TGR5 receptors are involved in various regulatory processes, ranging from the activation of macrophage to the regulation of glucose metabolism. BAs reduce the production of inflammatory cytokines through TGR5 activation in immune cells. BAs increase the energy expenditure of skeletal muscle and brown adipose tissue through iodothyronine deiodinase (D2), thereby preventing obesity (Castellanos-Jankiewicz et al., 2021). BAs regulate postprandial insulin release and blood glucose concentration by activating TGR5 in enteroendocrine L cells and stimulating the release of incretin GLP-1 (Kuhre et al., 2016). It has also been shown that TGR5 activation promotes glucose-induced insulin release from pancreatic β-cells in a cAMP- and calcium-dependent manner, meanwhile, avoiding glucagon release from α-cells (Figure 1) (Huang et al., 2019).

### 4.3 Bile acids promote insulin secretion by regulating GLP-1

Although only 1% enteroendocrine cells in the intestinal epithelium, the intestine is the largest endocrine organ due to its length. The enteroendocrine K cells secrete the glucose-dependent insulinotropic polypeptide (IP) to promote insulin release after food intake (Trabelsi et al., 2017). GLP-1 secreted by the enteroendocrine L cells, is a gastrointestinal hormone released in response to feeding that inhibits gastric emptying, appetite (Tomaro-Duchesneau et al., 2020), and glucagon secretion by exerting an incretin effect (Hira et al., 2020). After GLP-1 binds to the GLP-1 receptor on the islet β-cell membrane, it phosphorylates K^+^−ATPase. Consequently, the cell membrane is depolarized, K^+^ channels close, Ca^2+^ channels open, and Ca^2+^ influx stimulates insulin secretion from β-cells, resulting in the closure of K^+^ channels, the depolarization of the cell membrane, the opening of Ca^2+^ channels, and the influx of Ca^2+^, stimulates insulin secretion from β-cells. Meanwhile, by binding to the receptor, GLP-1 activates adenylate cyclase (AC) on the cell membrane, which converts ATP to cAMP. As a second messenger, cAMP activates protein kinase A (PKA) and cAMP-regulated guanine nucleotide exchange factor II (GEF II) to promote insulin secretion (Gomez et al., 2002).

BAs can regulate GLP-1 secretion and proglucagon gene expression. It was showed that both TGR5 and FXR were expressed in L cells. TGR5 is located on the basolateral of L cells, which mediated the BAs-induced GLP-1 secretion (Harach et al., 2012). The high-fat-fed TGR5 transgenic mice (TGR5-tg mice) exhibited improved glucose tolerance, whereas TGR5-KO mice fed with high fat diet had worse glycemic status. After oral glucose tolerance test (OGTT), TGR5-tg mice fed with high fat diet showed higher GLP-1 and insulin levels than TGR5-WT mice. It suggests that the improvement in glucose metabolism is achieved by GLP-1-mediated incretin action. TGR5/GLP-1 pathway is important in the improvement of blood glucose by BAs. Through TGR5 activation, BAs also initiate cAMP and its downstream related signaling pathways, type 2 iodothyronine deiodinase (D2), Ca^2+^/calcineurin-activated nuclear factor of activated T cell 3 (NFAT 3), proprotein convertases 1/3 (PC 1/3) (Morimoto et al., 2016), or mammalian target of rapamycin (mTOR) (Zhai et al., 2018), which stimulate the secretion of GLP-1 from intestinal L cells, promote insulin secretion (Cao et al., 2016; Lasalle et al., 2017), and improve the pancreatic β-cell function and blood glucose homeostasis, thereby improving T2DM. In addition, the activation of intestinal TGR5 also promotes the release of peptide tyrosine tyrosine (PYY), which reduces appetite (Bala et al., 2014).

Conversely, the activation of FXR inhibits the ileal proglucagon gene expression and GLP-1 secretion by inhibiting glycolysis and carbohydrate response element binding protein (ChREBP) activity in L cells (Trabelsi et al., 2015). The deletion of FXR gene improved glucose metabolism by enhancing peripheral glucose handling and increasing adipose tissue insulin sensitivity in hyperphagic ob/ob mice. Nevertheless, this effect was not observed in hepatocytes from ob/ob mice lacking the FXR gene (Prawitt et al., 2011), thus demonstrating the beneficial effect of FXR gene deletion in extrahepatic tissues on glucose homeostasis.

In conclusion, BAs have beneficial effects on T2DM by regulating GLP-1 to activate GPCRs, reducing food intake, improving insulin sensitivity, inhibiting fat accumulation, and reducing systemic inflammation. The reduction of BAs may reduce these beneficial effects and promote the development of IR and T2DM (Figure 1).

### 4.4 Bile acids improve insulin sensitivity by activating FGF15/19 and FGF21 pathways

**FIGURE 2:**
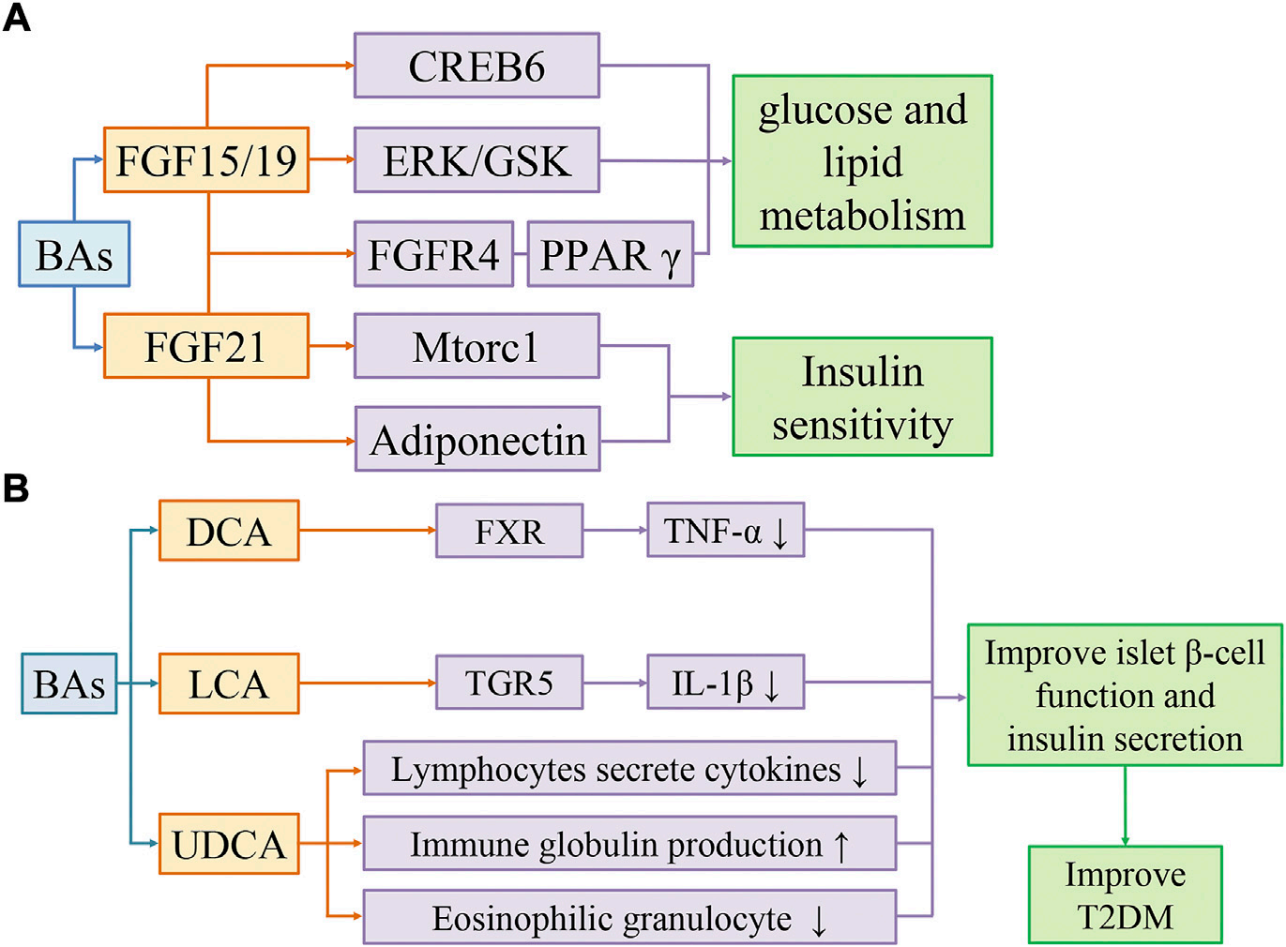
Bile acids activate FGF15/19 and FGF21 pathways to improve insulin sensitivity **(A)**. Bile acids improve T2DM by reducing inflammation **(B)**. **(A)** BAs improve glucose and lipid metabolism through FGF19/CREB6, FGF19/EPK/GSK, and FGF19/FGFR4/PPARγ pathways, and improve insulin sensitivity through FGF21/Mtorc1 and FGF21/Adiponectin pathways. **(B)** DCA activates FXR to reduce TNF-α levels. LCA activates TGR5 to reduce IL-1β levels. UDCA regulates the immune system by reducing cytokine secretion by lymphocytes, producing immunoglobulins, and inhibiting eosinophil activation and degranulation. Secondary bile acids can improve T2DM by restoring islet β-cell function and insulin secretion affected by inflammation.

The feeding-induced fibroblast growth factor 19 (FGF19) and the fasting-induced fibroblast growth factor 21 (FGF21) are metabolic regulatory hormones with different physiological functions, but both have similar effects in improving energy metabolism and insulin sensitivity (Kuro-O, 2019). The fibroblast growth factor 15 (FGF15) in rodents and the FGF19 in humans are highly expressed in ileal epithelial cells and can be secreted by BAs-activated FXR or TGR5, which is an important endocrine hormone that regulates hepatic bile acid synthesis and host glucose and lipid metabolic homeostasis (Somm and Jornayvaz, 2018). FGF15 can affect the peroxisome proliferator-activated receptor *γ* (PPARγ) pathway through hepatic fibroblast growth factor receptor 4 (FGFR4), reducing fat accumulation and cholesterol, triglyceride synthesis. FGF15 and FGF19 can reduce hepatic gluconeogenesis by regulating the dephosphorylation of the gluconeogenic transcription factor cAMP responsive element binding protein 6 (CREB6) (Potthoff et al., 2011). On the other hand, they activate the phosphorylation cascade of extracellular signal-regulated kinase (ERK)-glycogen synthase kinase a/p (GSK a/p) to promote hepatic glycogen synthesis (Kir et al., 2011). In hypothalamic neurons, FGF15 or FGF19 activates the ERK signaling pathway to reduce insulin-independent glucose levels (Lan et al., 2017; Liu et al., 2018). In addition, they can increase metabolic rate and reduce body weight by increasing β⁃Klotho-dependent sympathetic activity in brown adipose tissue (Lan et al., 2017). The plasma levels of FGF19 were significantly decreased in T2DM and obese patients, while injection of recombinant FGF19 protein significantly improved metabolic disorders in db/db mice and DIO mice (Guo et al., 2022). FGF21 is a nutrient-sensitive hormone produced in hepatocytes, which can be induced by BAs. FGF21 improves insulin sensitivity through several mechanisms. In the liver, FGF21 inhibits the mammalian target of rapamycin complex 1 (Mtorc1) signaling pathway and increases hepatic insulin sensitivity (Pathak and Chiang, 2019). In adipose tissue, FGF21 promotes fatty acid oxidation by activating PPARγ, thereby improving insulin sensitivity (Wu et al., 2020). FGF21 also stimulates adiponectin (APN) secretion in adipose tissue, thereby reducing ceramide and blood glucose, and enhancing insulin sensitivity in DIO mice (Figure 2A) (Holland et al., 2013).

### 4.5 Bile acids improve T2DM by reducing the levels of inflammatory cytokines

In individuals with obesity, T2DM and MAFLD, the chronic low-grade inflammation is usually accompanied by metabolic changes in tissues and organs, which is characterized by recruitment of immune cells, abnormal production of cellular inflammatory cytokines and acute phase reactants, and activation of inflammasome (Weichhart et al., 2015). In inflammatory states, gut microbiota converts bile salts to BAs and SBAs inefficiently (Lloyd-Price et al., 2019). Compared with healthy subjects, patients with IBD have an imbalance of gut microbiota, lower concentrations of SBAs and higher concentrations of conjugated bile acids in feces and blood circulation (Duboc et al., 2013).

BAs inhibits the induction of pro-inflammatory genes by inhibiting the nuclear factor kappa-light-chain-enhancer of activated B cells (NF-κB) pathway (de Aguiar Vallim et al., 2013). SBAs such as DCA and LCA can inhibit TNF-α production in macrophages through FXR. FXR^−/−^ mice showed an increased risk of colitis and cancer compared to wild-type mice (Renga et al., 2013). SBAs, especially LCA, inhibited IL-1β production in macrophages *via* TGR5 (Guo et al., 2016). The clinical trials have shown that the elevated IL-1β in circulating system is a risk factor for T2DM, so the inhibition of IL-1β may be a promising therapeutic strategy (Zhao et al., 2014). Furthermore, SBAs can block the hyperactivation of the inflammasome in T2DM (Guo et al., 2016). Gut microbiota such as *Ruminococcus* can synthesize UDCA. As a SBA, UDCA can regulate the immune system by reducing cytokine secretion by lymphocytes, producing immunoglobulin, and inhibiting eosinophil activation and degranulation (Hosseinkhani et al., 2021).

TGR5 and FXR are expressed in a variety of immune cells as two major receptors for BA. TGR5 has anti-inflammatory effects and reduces the production of inflammatory cytokines in monocytes, macrophages, and dendritic cells (Hu et al., 2022). Activated TGR5 inhibits NLRP3 inflammasome activation *via* the TGR5-cAMP-PKA axis, thereby attenuating high-fat diet-induced elevated glucose tolerance, IR, and inflammatory responses (Hu et al., 2021). Secondly, the activation of TGR5 by BA can reduces the CCAAT/enhancer binding protein *ß* (C/EBPβ)-mediated inflammatory infiltration of macrophages in white adipose tissue and increase energy expenditure (Velazquez-Villegas et al., 2018). On the other hand, FXR deficiency impairs intestinal barrier function, which may enhance hepatic LPS secretion and inflammatory responses.

Overall, BAs have multiple signaling regulatory roles in regulating the immune system and inflammation. And the low-grade inflammation has been considered as a potential cause of IR. Adipose tissue itself releases many cytokines. Therefore, as the increase of adipose tissue is accompanied by the accumulation of various metabolic stresses, the cytokines released from adipose tissue will “overflow”, resulting in an imbalance between insulin-sensitive cytokines (adiponectin, leptin) and pro-inflammatory cytokines (RBP4, resistin, IL-6, TNF). However, it is worth noting that IR can also occur without an inflammatory response. The studies found that rats and humans exhibited IR before an inflammatory response (Roden and Shulman, 2019). Following the intervention of dietary palm oil (PO) composed of saturated fatty acids, the hepatic γATP and HCL concentrations were increased in mice, which simultaneously induced lipid oxidation and IR, and the human subjects developed systemic IR. The plasma free fatty acid (FFA) concentrations were significantly increased in humans and mice after PO intake, but there were no changes in circulating inflammatory markers or adipokines, such as TNF-α, IL-6, and fetuin-A (Figure 2B) (Hernández et al., 2017).

### 4.6 Effects of 12α-hydroxylated bile acids and non-12α-hydroxylated bile acids on insulin sensitivity

Insulin is the only hormone in the body that reduces blood glucose and promotes the synthesis of glycogen, fat, and protein simultaneously, which plays a crucial role in glucose and lipid metabolism. Abnormal insulin sensitivity is one of the important reasons for the disorder of glucose and lipid metabolism *in vivo*. Several studies analyzed the relationship between bile acid concentrations and insulin sensitivity. The results showed that IR was associated with the concentrations of primary bile acids and 12α-hydroxylated bile acids (Haeusler et al., 2013; Legry et al., 2017).

12α hydroxylated bile acids are mainly catalyzed by cytochrome P450 family 8 subfamily B member 1 (Cyp8b1). The plasma concentrations of 12α-hydroxylated bile acids were higher in obese and T2DM patients than in healthy people (Kaur et al., 2015). In DIO mice, knockdown of the Cyp8b1 gene reduced body weight, decreased postprandial insulin levels, and improved glucose tolerance, despite no changes in thermogenesis and oxygen consumption. The fecal lipid content was higher in Cyp8b1 KO mice indicated that the lipid absorption capacity was reduced in the intestine. The number of ileal L cells was increased and the contents of GLP-1 and PYY were increased in the ileal epithelium in Cyp8b1 KO mice, which may be due to impaired lipid absorption and lipid receptors activation. The lipidomic results showed that knockdown of Cyp8b1 increased the excretion of fecal lipids such as monoacylglycerol, which is an activator of the lipid receptor G protein coupled receptor 119 (GPR119). GPR119 is mostly distributed on the surface of enteroendocrine cells, especially L cells, and mediates lipid-induced GLP-1 secretion. Cyp8b1 KO mice had higher levels of ileal GLP-1 and PYY contents, slowed gastric emptying, and decreased feed intake and body weight, which could be relieved by knocking out GPR119. The inefficient hydrolysis of triglyceride in Cyp8b1 KO mice results in the entry of unabsorbed lipids into the small intestine and colon, which activates GPR119 to induce the release of GLP-1 and PYY, slow gastric emptying, and reduce food intake (Higuchi et al., 2020). Thus, changes in the content of 12α-hydroxylated bile acids can affect lipid absorption, intestinal hormone secretion, and lipid receptor activation, thereby affecting insulin sensitivity, and glucose and lipid metabolism. In addition, 12α-hydroxylated bile acids also inhibit hepatic PPARα-FGF21 signaling pathway by activating intestinal FXR to generate ceramide and FGF15, thus leading to IR (Chiang and Ferrell, 2019; Pathak and Kakiyama, 2019).

BAs can regulate intestinal lipid receptors in multiple ways. It has been proved that BAs promotes the hydrolysis and absorption of dietary fat, which is an endogenous mechanism to prevent satiety induction by enteroendocrine cells (Higuchi et al., 2020). The bile acid composition of Cyp8b1 KO mice is altered with a decrease in 12α-hydroxylated bile acids and an increase in non-12α-hydroxylated bile acids, which induces impaired jejunal oleoylethanolamide production, slow gastric emptying, and increased satiety after feeding (Haeusler et al., 2013). The non-12 hydroxylated bile acids resist the obesity phenotype in mice through TGR5-mediated activation of brown adipose tissue (BAT) and upregulation of uncoupling protein 1 (UCP1) expression (Wei et al., 2020). Dieting significantly reduced the proportion of non-12α-hydroxylated secondary bile acids such as UDCA and LCA in mice. Calorie restriction (CR) diets have been recognized as a factor in promoting health, prolonging lifespan, and preventing the development of metabolic diseases (Kökten et al., 2021). However, long-term weight loss is challenging due to the complex interaction between hormones and behavior (Liu et al., 2022), which often results in gradual or rapid weight regain after CR intervention (Marlatt et al., 2017). Recently, study by Mengci Li showed that the beneficial bacteria *Parabacteroides distasonis* in mice was significantly reduced during fasting or CR. The supplementation of non-12α hydroxylated bile acids UDCA and *Parabacteroides distasonis* to high-fat diets prevented weight gain and improved glucose and energy metabolism. Mice can reduce weight rebound by supplementation with non-12α hydroxylated bile acids or *Parabacteroides distasonis* (Li et al., 2022).

### 4.7 Probiotic treatment improve T2DM by regulating Cyp8b1 gene

Probiotic metabolites regulate the expression and activity of intestinal nuclear receptors (NRs), including VDR, FXR, and PPARs, whose activity extends to the cerebral cortex (Distrutti et al., 2014). Regulation of gut microbiota by administration of the probiotic cocktail VSL#3 increased fecal bile acid excretion in mice, as well as *de novo* hepatic bile acid synthesis, associated with increased BSH transcription and fecal enzyme activity. VSL#3 is a probiotic formulation consisting of *Lactobacillus acidophilus, Lactobacillus paracasei, Lactobacillus bulgaricus, Lactobacillus plantarum, Bifidobacterium breve, Bifidobacterium infantis, Bifidobacterium longum, and Streptococcus thermophilus*. VSL#3 significantly altered the composition of fecal microbiota in mice administered by gavage at a dose of 50 × 10^9^ CFU/d for 21 days, increasing the number of *Firmicutes* and *Actinomycetes*, while decreasing *Bacteroides,* and *Bacillus Proteus*. VSL#3 increases the expression of Cyp7a1 gene by down-regulating the expression of FGF15 in the ileum, activates GPCR, promotes the secretion of GLP-1, and increases insulin sensitivity (Supplementary Table S1) (Jena et al., 2020).

### 4.8 Future directions and therapeutic approaches for T2DM based on the bile acids signaling

The incidence of T2DM is increasing worldwide. T2DM patients required long-term drug treatment with the high risk of cardiovascular disease and other complications. T2DM caused a huge medical burden to individuals and society. Therefore, there is an urgent need to explore effective approaches to prevent and improve blood glucose and blood lipid levels in T2DM patients. At present, the improvement of T2DM based on the interaction of BAs and gut microbiota shows the prospect of effective intervention through bile acid signaling in the future (Massey and Brown, 2021). The potential targeting signaling pathways and targets of BAs and their derivatives in improving T2DM include: FGF15/19 regulates hepatic glucose and lipid metabolism by PPARγ, ERK signaling pathways and CREB6 dephosphorylation (Potthoff et al., 2011); FGF21 enhances insulin sensitivity by Mtorc1 signaling pathway (Pandak and Chiang, 2019) and stimulating APN secretion (Holland et al., 2013). The results suggest that fibroblast growth factor and its related pathways contribute to glycemic control and reduce lipid synthesis and accumulation, which may become a worthy intervention on T2DM. In addition, BAs also reduce the secretion of inflammatory cytokines such as TNF-α (Renga et al., 2013) and IL-1β (Guo et al., 2016), improve the function of islet β-cells, and increase insulin secretion by regulating TGR5 and FXR in the liver and intestine (Cao et al., 2016; Lasalle et al., 2017). Studies have shown that FXR antagonists and TGR5 agonists in the intestine can improve glycemic control in rodents with T2DM (Zheng et al., 2021). TGR5 and FXR become potential targets for reducing insulin resistance and improving T2DM. Therefore, the development of gut-specific FXR antagonists and TGR5 agonists may be a promising approach for the treatment of hyperglycemia, but the long-term impact of this approach needs to be evaluated (Shapiro et al., 2018).

## 5 Therapeutic effects of bile acids and their derivatives on type 2 diabetes mellitus

### 5.1 Obeticholic acid

Obeticholic acid (OCA) is a derivative of the primary bile acid CDCA and a selective FXR agonist with anti-cholestatic activity and inhibit intestinal inflammation (Fiorucci et al., 2011). In animal studies, OCA was able to increase insulin sensitivity, inhibit gluconeogenesis and adipogenesis, and have anti-inflammatory and anti-fibrotic effects. OCA also ameliorated high-fat diet-induced obesity and IR in mice, and fatty liver and IR in Zucker (fa/fa) rats. OCA can antagonize NF-κB-stimulated liver inflammation, regulate innate immunity in animal models of colitis, inhibit IBD, and protect intestinal barrier (Guo et al., 2021). OCA has entered phase II clinical trials in patients with type 2 diabetic metabolic fatty liver disease, with improvements in insulin sensitivity, hepatic gamma-glutamyl transferase levels, and weight loss (Mudaliar et al., 2013).

### 5.2 6α-ethyl-23(S)-methyl cholic acid, INT777

The TGR5 specific agonist 6α-ethyl-23(S)-methyl-CA (EMCA or INT-777) is a CA derivative. In animal model, EMCA improved glucose tolerance, stimulated GLP-1 secretion from enteroendocrine L cells, improved insulin sensitivity, and increased the intracellular ATP/ADP ratio in obese mice. TGR5 agonists can reduce intestinal inflammation and prevent liver inflammation (Wu et al., 2019). Activation of TGR5 also reduced macrophage inflammation and lipid load to reduce atherosclerotic lesions (Li et al., 2018).

### 5.3 Bile acid sequestrant

Bile acid sequestrant (BAS) is an anion exchange resin originally used to reduce hypercholesterolemia. BAS increased HDL-cholesterol levels by 3%–5% and reduced LDL-cholesterol levels by 15%–30%, while serum triacylglycerol (TAG) levels were unchanged or increased slightly. BAS reduces intestinal FXR activity by trapping BA in the intestinal lumen and increasing BA excretion in the feces. Cholesterol continues to be converted by hepatocytes into bile acids, lowering plasma cholesterol levels (Hao et al., 2017).

BAS stimulates GLP-1 secretion by inhibiting ileal reabsorption of BAs. BAS drives BAs to the colon where L-cell density is highest. L-cell density is greatest in the colon, where BAs are driven by the BAS. BAs bind and activate TGR5 on L-cell, reducing intestinal glucose absorption, and promoting hepatic glycolysis and lipogenesis (Harach et al., 2012). Further study showed that BAS improved blood glucose by reducing glycogenolysis in the liver *via* TGR5/GLP-1 (Potthoff et al., 2013). The effects of colsevelam hydrochloride on blood glucose homeostasis were observed only in FXR ob/ob WT mice, but not in FXR KO ob/ob mice, suggesting that FXR may be responsible for the effects of Colesevelam hydrochloride (Supplementary Table S1) (Hartmann et al., 2022). It is consistent with these results that BAS treatment improves glucose clearance and insulin sensitivity in T2DM patients. After taking Colesevelam hydrochloride, insulin secretion increased. Long-term treatment of T2DM obese ZSF1 rats with another BAS, SAR442357, increased plasma insulin levels and decreased HbA1c, cholesterol, and triglyceride levels (Supplementary Table S1). Unlike Colesevelam hydrochloride, SAR442357 also slows the progression of diabetic kidney disease (DKD) (Castañeda et al., 2021).

### 5.4 Tauroursodeoxycholic acid

Tauroursodeoxycholic acid (TUDCA) can activate FXR in mouse pancreatic β-cells, inhibit the activity of sulphonylurea receptor 1 (SUR1, a subunit of ATP-sensitive potassium channel), reduce K^+^ efflux, increase cytoplasmic Ca^2+^ concentration, and promote insulin secretion. And the activation of TGR5 in islet α-cells can induce the expression of glucagon proconvertase-1 (PC1), which catalyzes the production of GLP-1 from glucagon. Then GLP-1 migrates to β-cells and binds to the GLP-1 receptor on its membrane to enhance the insulin secretion function of β-cells (Kumar et al., 2016). Recent studies have found that TUDCA affects blood glucose homeostasis and insulin signaling by activating insulin receptors. TUDCA intervention improves glucose homeostasis in DIO mice, which is eliminated by injection of the insulin receptor antagonist S961. Molecular docking showed that TUDCA exhibited high affinity for TGR5 and insulin receptor and interacted strongly with insulin binding sites one and two of insulin receptor (Supplementary Table S1) (da Silva et al., 2021; Zangerolamo et al., 2021).

### 5.5 Hyodeoxycholic acid

Hyodeoxycholic acid (HCA) accounts for 80% of porcine bile. The trace amounts of HCA and its derivatives have been found in human blood (Spinelli et al., 2016). Pigs are unique model animal with a diabetic lifestyle but are resistant to developing T2DM. *In vitro* HCA upregulates GLP-1 secretion and preproglucagon gene (Gcg) transcription. HCA is found to act as both a TGR5 agonist and an FXR antagonist to promote GLP-1 secretion and Gcg gene transcription in mice, which is a unique mechanism not found in other bile acid producing species. The effects of HCA on serum GLP-1 and insulin levels, glucose tolerance, and insulin tolerance in TGR5^+/+^ mice were significantly eliminated in TGR5^−/−^ mice, suggesting that HCA upregulates GLP-1 secretion *via* TGR5 *in vivo*. In a clinical cohort study, it was confirmed that lower concentrations of serum HCA were associated with diabetes and closely correlated with glycemic markers, suggesting a protective effect of HCA on the development of diabetes in mammals and may be used for T2DM treatment (Zheng et al., 2021).

### 5.6 Ursodeoxycholic acid

As the drug of choice for the treatment of primary biliary cirrhosis and the dissolution of cholesterol stones, UDCA mainly regulates the physiological function of cholesterol by reducing the intestinal absorption speed of cholesterol and breaking down cholesterol-containing micelles (Bellentani, 2005). In liver disease, UDCA provides clinical benefits by mediating hepatocyte protection, bile secretion and immunomodulation. The hepatocyte-protective effect of UDCA is partly derived from the inhibition of apoptosis (Lukivskaya et al., 2007). UDCA also reduces the permeability of mitochondrial membranes and the release of hydrolases from damaged hepatocytes (Quintero et al., 2014). UDCA was found to improve hepatic steatosis and insulin sensitivity by inducing hepatic lipid excretion and inhibiting hepatic long-chain free fatty acid uptake in obese mice (Nie et al., 2012; Castro et al., 2013; Oh et al., 2016). The effect of UDCA on age-related body fat accumulation and the anti-obesity mechanisms of UDCA in the liver and white adipose tissue was investigated in humans. The results showed that long-term administration of UDCA reduced hepatic lipogenesis and inflammatory responses, which supported the beneficial role of UDCA in the prevention and treatment of T2DM (Al-Salami et al., 2017).

UDCA has anti-inflammatory effects on the ileal mucosa and increases the survival rate of pancreatic β-cells (Mooranian et al., 2016), which suggests its potential application value in T2DM. Three weeks after UDCA intervention was conducted in diabetic Balb/c mice, the blood, tissue, urine, and feces were collected for blood glucose, inflammation, and bile acid analysis. The results showed that UDCA had a regulatory effect on bile acid composition, but did not affect blood glucose levels. The concentrations of blood glucose, HbA1c, HOMA-IR, IFN-γ, IL-6, and IL-10 in T2DM mice remained similar to those in control group, suggesting that UDCA lacked significant hypoglycemic or anti-inflammatory effects in T2DM mice. The levels of CDCA, LCA and UDCA in plasma, brain, and skeletal muscle of T2DM mice were significantly increased after UDCA intervention, suggesting that UDCA regulates bile acid composition and enterohepatic cycling, and may treat T2DM by changing the concentrations of other BAs such as LCA and UDCA (Supplementary Table S1) (Mooranian et al., 2022).

### 5.7 Lithocholic acid

LCA is a second endogenous agonist of the vitamin D receptor (VDR) (Sasaki et al., 2021). LCA can prevent apoptosis of mouse colonic epithelial cells to maintain intestinal barrier function. LCA was found to be more effective than UDCA in preventing the release of TNF-α from colonic epithelial cells *in vitro*. LCA was also found to be more efficient than UDCA in reducing dextran sodium sulfate-induced inflammation in mice with colitis in animal experiments. In animal experiments, LCA was also more effective than UDCA in preventing dextran sulfate sodium (DSS)—induced inflammation in mice with colitis. The body weight was significantly reduced by LCA treatment, which may be due to the reduced food intake in LCA-treated mice or the effects of BAs on energy expenditure and fat metabolism (Schoeler and Caesar, 2019).

## 6 Other treatments for Type 2 diabetes mellitus

### 6.1 Vertical sleeve gastrectomy

Bariatric surgery is one of the most effective and long-lasting treatments for pathologic obesity, T2DM and fatty liver disease (Mingrone et al., 2015). Vertical Sleeve Gastrectomy (VSG) is the most common procedure in bariatric surgery (Angrisani et al., 2015; Buwen et al., 2015). Elevated GLP-1 in circulatory system and changes in systemic BAs level were continuously observed after surgery (Chambers et al., 2014). VSG significantly alters the production, secretion, and redistribution of BAs. A study analyzed the factors that led to the decrease of intestinal BAs levels in obese mice after VSG surgery: on the one hand, the mRNA and protein expression levels of the bile salt export pump were significantly reduced, which reduced the size of the intestinal bile acid pool; on the other hand, VSG-induced increase in intestinal permeability or loss of FXR activity may also lead to decreased BAs levels by promoting paracellular absorption in the proximal intestine. VSG reduces intestinal BAs levels, which leads to impaired intestinal lipid absorption. This mechanism of caloric intake reduction may contribute to the metabolic improvement (Ding et al., 2021a). Another study indicated an increase in endogenous cholic acid-7-sulfate (CA7S) in the gastrointestinal tract of mice and humans after VSG (Supplementary Table S1). As a TGR5 agonist, CA7S regulates blood glucose by increasing TGR5 activation and GLP-1 secretion (Chaudhari et al., 2021).

### 6.2 Takeda G-protein receptor 5 agonists and farnesoid X receptor inhibitors

It has been shown that HCA can promote GLP-1 secretion and improve blood glucose homeostasis by activating TGR5 and inhibiting FXR (Zheng et al., 2021), suggesting that the development of TGR5 agonists and FXR antagonists is an effective way to treat T2DM. As a promising drug target, TGR5 can be used to screen new therapeutic agents for T2DM (Velazquez-Villegas et al., 2018), but the application of TGR5 agonists has been limited in recent years due to systemic side effects, such as gallbladder emptying (Stepanov et al., 2013). Recent studies have shown that it is possible to develop a more selective TGR5 agonist. A TGR5 agonist, RDX8940, improved hepatic steatosis and insulin sensitivity without systemic side effects in mice model of MAFLD (Supplementary Table S1) (Finn et al., 2019).

Because that TGR5 agonists absorbed into the circulation would cause significantly off-target effects, intestinal-restricted TGR5 agonists have been proposed as a potential improved therapy for T2DM (Cao et al., 2016). CA7S is an intestinal-restricted TGR5 agonist that increases TGR5 expression both *in vitro* and *in vivo*. Similarly, it can induce intestinal endocrine L cells to secrete insulin hormone GLP-1 both *in vitro* and *in vivo*. It has shown that antidiabetic effects were found under both acute and chronic conditions on lowering blood glucose levels and improving glucose tolerance in insulin-resistant mice. Unlike other known endogenous TGR5 agonists, CA7S is not absorbed by the portal vein or systemic circulation. Furthermore, CA7S does not transport across the intestinal epithelium by active or passive way. It also does not impair the integrity of the epithelial cell barrier. Due to its beneficial metabolic effects, intestinal restriction, and low toxicity, CA7S can be a drug candidate for T2DM (Chaudhari et al., 2021).

Ginsenosides is the main active components of ginseng, which regulates glucose and lipid metabolism by inducing gluconeogenesis reduction and glucose transport (Bai et al., 2018). Ginsenoside Ro improves IR by activating TGR5 to promote GLP-1 secretion and energy expenditure in adipose tissue in DIO mice, which suggests that ginsenoside Ro is a potential compound for the treatment of T2DM (Supplementary Table S1) (Jiang et al., 2021). Jyoti et al. found that Pregnane-Oximino-Alkyl-Amino-Ether (compound 14b), a TGR5 agonist, improved glucose tolerance in sucrose-loaded rats by increasing glucose utilization in skeletal muscle and hepatocytes (Supplementary Table S1). It also showed anti-hyperglycemic activity in STZ-induced diabetic rats and genetically diabetic db/db mice (Gupta et al., 2022), It is suggested that compound 14b can be used as a leading drug in the treatment of T2DM and related metabolic complications.

Notoginsenoside Ft1 (Ft1) extracted from *Panax notoginseng* is identified as an effective TGR5 ligand *in vitro*. Ft1 is not only a TGR5 agonist but also an FXR antagonist. Ft1 can alleviate IR in obese mice. On the one hand, Ft1 activates intestinal TGR5 to promote intestinal GLP-1 release. On the other hand, Ft1 upregulates CYP7A1 and CYP27A1 expression in the liver by inhibiting transcriptional activation of intestinal FXR. CYP7A1 and CYP27A1 are the two key enzymes in the classical and alternative pathways of BAs synthesis respectively, whose high expression increases hepatic BAs synthesis. The elevated serum levels of BAs subsequently activate TGR5 in adipose tissue to increase energy expenditure (Ding et al., 2021b). Therefore, Ft1 may be a novel compound with opposite effects on two key bile acid receptors, which could serve as a potential lead compound for antidiabetic drugs. In addition, the TGR5/FXR dual agonist, 6α-ethyl-24-nor-5β-cholane-3α,7α,23-triol-23 sulfate sodium salt (INT-767), significantly inhibits high-fat diet-induced hepatic steatosis in mice, restores insulin sensitivity, and induces differentiation of preadipocyte to a metabolically healthy phenotype (Comeglio et al., 2018).

### 6.3 Apical sodium-dependent bile acid transporter inhibitors

Apical sodium-dependent bile acid transporter inhibitors (ASBT-I) reduce intestinal BAs reuptake by inhibiting ASBT. ASBT-I can improve insulin sensitivity in mice fed with high-fat diet. Similar to BAS, ASBT-I reduces intestinal BAs absorption, reduces hepatic BAs supply, activates FXR, de-inhibits CYP7A1, and promotes the conversion of cholesterol to BAs. Furthermore, translocation of BAs to the distal end of the intestine may maintain the secretion of TGR5-induced GLP-1. Compared with BAS, ASBT-I treatment does not impair the absorption of free BAs (Rao et al., 2016).

### 6.4 Alpha-glucosidase inhibitor

Alpha-glucosidase inhibitor (AGI) is an oral hypoglycemic drug which was frequently used in Asian populations. AGI can reduce postprandial glucose level by inhibiting polysaccharide digestion (Qiu et al., 2020). Recently AGI has been shown to significantly affect gut microbiota and interfere with its bile acid metabolism. AGI can improve IR and β-cell function by regulating bile acid metabolism of gut microbiota to affect host BAs signaling, and synergistically promote its hypoglycemic and hypolipidemic effects (Qiu et al., 2020). Similar changes were found with metformin, a more widely used oral hypoglycemic drug (Wu et al., 2017; Sun et al., 2018). Since the regulation of the host bile acid composition is critical, the role of gut microbiota on bile acid metabolism in mediating the therapeutic effect of oral hypoglycemic drugs or in the management of T2DM needs to be further elucidated.

### 6.5 Docosahexaenoic acid/eicosapentaenoic acid

The replacement dietary fat by n-3 polyunsaturated fatty acids can effectively prevent hyperglycemia in diet-induced obesity (DIO) mice (Flachs et al., 2014). It was found that supplementation of docosahexaenoic acid (DHA)/eicosapentaenoic acid (EPA) could significantly reduce hyperglycemia and IR and restore glucose homeostasis in db/db mice. DHA/EPA can reduce β-cell apoptosis, inhibit hepatic gluconeogenesis, and promote GLP-1 secretion by changing gut microbiota composition and bile acid metabolism (Zhuang et al., 2021). DHA/EPA regulates the composition of gut microbiota and increases its diversity, reducing the number of *Enterobacteriaceae* with LPS and pathogenic bacteria such as *Staphylococcus*, *Streptococcus,* and *Klebsiella* (Blin et al., 2017; Younge et al., 2017). DHA/EPA can also change the bile acid composition, increase the primary bile acids CDCA and CA, decrease TCA, activate FXR-SHP-FOXO1 pathway, and inhibit hepatic gluconeogenesis (Zhuang et al., 2021). DHA/EPA can be an effective therapeutic dietary supplement for T2DM patients.

### 6.6 Exercise improves bile acid metabolism by regulating gut microbiota

Healthy eating habits and moderate physical activity contribute to improving insulin sensitivity, lipid metabolism, and hepatic steatosis in obese patients (Ordonez et al., 2015). In adults and animal models, the benefits of exercise on metabolism are associated with its ability to reshape gut microbiota composition, suggesting that exercise may be a potential strategy to improve metabolic health by regulating gut microbiota. Activation of FXR-dependent pathways promotes metabolic disease in diet-induced and genetic obese rodents (Gonzalez et al., 2016). The mechanism involves microbiota imbalance, which is due to its ability to alter bile acid pools and signaling properties (Arab et al., 2017). Exercise can effectively inhibit the imbalance of gut microbiota caused by high-fat diet, thereby maintaining the intestinal barrier, promoting the enterohepatic cycling, and improving bile acid homeostasis. Exercise also downregulates the expression of hepatic and intestinal bile acid transporters and reduces FXR induction and SHP overexpression to restores high-fat diet-induced imbalance in bile acid metabolism (Carbajo-Pescador et al., 2019).

## 7 Conclusion

T2DM is a chronic metabolic disease with multiple pathogenesis that affects not only the liver but almost all other organs of the body. As a multi-risk factor disease, T2DM needs a multiple-effect therapy to treat hyperglycemia, IR, lipid metabolism disorder, inflammation, and hepatic fibrosis. As a potential therapeutic target, the regulation of bile acid composition is one of the main physiological functions of gut microbiota. Diverse BAs are synthesized under the action of gut microbiota. At present, the role of these signaling molecules produced in the bile acids-gut microbiota crosstalk is only partially confirmed. There is still some disagreement regarding the overall role of TGR5 and FXR in glucose metabolism, but no doubt that these receptors play a crucial role in maintaining glucose homeostasis in the intestine. The activation of FXR in hepatocytes improves glucose metabolism. Glucose clearance and insulin sensitivity may be better enhanced by the inhibition of FXR in the intestine. In enteroendocrine L cells, TGR5 activation increases insulin and GLP-1 release. Conversely, FXR activation caused a decrease in glucose-induced GLP-1 production. In conclusion, bile acids and their derivatives have a clear therapeutic effect on T2DM. The blood glucose homeostasis can be improved by regulating bile acid metabolism. A better understanding of the bile acids-gut microbiota interaction is beneficial for the prevention and treatment of T2DM.

## Glossary

**T2DM**: type 2 diabetes mellitus
**IR**: insulin resistance
**BAs**: bile acids
**SBAs**: secondary bile acids
**GPCRs**: G protein coupled receptors
**CYP7A1**: cholesterol 7α-hydroxylase
**LXRα**: liver X receptor *aα*
**CDCA**: chenodeoxycholic acid
**CA**: cholic acid
**FXR**: farnesoid X receptor
**TGR5**: Takeda G-protein receptor 5
**CYP27A1**: sterol-27-hydroxylase
**CYP7B1**: oxysterol-7α-hydroxylase
**CYP7A1**: cholesterol-7α-hydroxylase
**ASBT**: apical sodium-dependent bile acid transporter
**ASBT-I**: apical sodium-dependent bile acid transporter inhibitors
**T-αMCA**: Tauro-α muricholic acid
**T-βMCA**: Tauro-β muricholic acid
**UDCA**: ursodeoxycholic acid
**LCA**: lithocholic acid
**VDR**: vitamin D receptor
**DSS**: dextran sulfate sodium
**DCA**: deoxycholic acid
**SCFAs**: short-chain fatty acids
**BSH**: bile salt hydrolase
**RCT**: reverse cholesterol transport
**TBA**: taurine conjugated bile acid
**FGF15**: fibroblast growth factor 15
**Akk**: Akkermansia muciniphila
**LPS**: lipopolysaccharide
**LBP**: lipopolysaccharide-binding protein
**IBD**: inflammatory bowel disease
**S-TBAs**: serum total bile acids
**PXR**: pregnane X receptor
**SXR**: xenobiotic receptor
**CAR**: constitutional androstane receptor
**S1PR2**: sphingosine-1 phosphate receptor 2
**SREBP-1c**: sterol regulatory element binding transcription factor-1c
**APOC**: apolipoprotein C
**ANGPTL3**: angiopoietin-like protein 3
**PPARα**: peroxisome proliferator-activated receptor α
**ABCG5**: ATP-binding cassette transport G5
**ABCG8**: ATP-binding cassette transport G8
**PEPCK**: phosphoenolpyruvate carboxykinase
**G6P**: glucose-6-phosphatase
**cAMP**: cyclic adenosine monophosphate
**GLP-1**: glucagon-like peptide 1
**IP**: insulinotropic polypeptide
**AC**: adenylate cyclase
**PKA**: protein kinase A
**GEF II**: guanine nucleotide exchange factor II
**OGTT**: oral glucose tolerance test
**D2**: type 2 iodothyronine deiodinase
**NFAT 3**: nuclear factor of activated T cell 3
**PC 1/3**: proprotein convertases 1/3
**mTOR**: mammalian target of rapamycin
**PYY**: peptide tyrosine tyrosine
**ChREBP**: carbohydrate response element binding protein
**FGF19**: fibroblast growth factor 19
**FGF21**: fibroblast growth factor 21
**PPARγ**: peroxisome proliferator-activated receptor γ
**FGFR4**: fibroblast growth factor receptor 4
**CREB6**: cAMP responsive element binding protein 6
**ERK**: extracellular signal-regulated kinase
**GSK a/p**: glycogen synthase kinase a/p
**APN**: adiponectin
**NF-κB**: nuclear factor kappa-light-chain-enhancer of activated B cells
**C/EBPβ**: CCAAT/enhancer binding protein β
**PO**: palm oil
**FFA**: free fatty acid
**Cyp8b1**: cytochrome P450 family 8 subfamily B member 1
**GPR119**: G protein coupled receptor 119
**BAT**: brown adipose tissue
**UCP1**: uncoupling protein 1
**CR**: calorie restriction
**NRs**: nuclear receptors
**OCA**: obeticholic acid
**EMCA**: 6α-ethyl-23(S)-methyl-CA
BAS: bile acid sequestrant
**TAG**: triacylglycerol
**DKD**: diabetic kidney disease
**TUDCA**: tauroursodeoxycholic acid
**SUR1**: sulphonylurea receptor 1
**PC1**: proconvertase-1
**HCA**: Hyodeoxycholic acid
**Gcg**: preproglucagon gene
**VSG**: Vertical Sleeve Gastrectomy
**CA7S**: cholic acid-7-sulfate
**AGI**: alpha-glucosidase inhibitor
**DIO**: diet-induced obesity
**DHA/EPA**: docosahexaenoic acid/eicosapentaenoic acid

## References

[B1] Ahmad T. R. Haeusler R. A. (2019). Bile acids in glucose metabolism and insulin signalling - mechanisms and research needs. Nat. Rev. Endocrinol. 15, 701–712. 10.1038/s41574-019-0266-7 31616073 PMC6918475

[B2] Al-Salami H. Mamo J. C. Mooranian A. Negrulj R. Lam V. Elahy M. (2017). Long-term supplementation of microencapsulated ursodeoxycholic acid prevents hypertension in a mouse model of insulin resistance. Exp. Clin. Endocrinol. Diabetes 125, 28–32. 10.1055/s-0042-106084 27219878

[B3] Angrisani L. Santonicola A. Iovino P. Formisano G. Buchwald H. Scopinaro N. (2015). Bariatric surgery worldwide 2013. Obes. Surg. 25, 1822–1832. 10.1007/s11695-015-1657-z 25835983

[B4] Arab J. P. Karpen S. J. Dawson P. A. Arrese M. Trauner M. (2017). Bile acids and nonalcoholic fatty liver disease: Molecular insights and therapeutic perspectives. Hepatology 65, 350–362. 10.1002/hep.28709 27358174 PMC5191969

[B5] Aviello G. Singh A. K. O'Neill S. Conroy E. Gallagher W. D'Agostino G. (2019). Colitis susceptibility in mice with reactive oxygen species deficiency is mediated by mucus barrier and immune defense defects. Mucosal Immunol. 12, 1316–1326. 10.1038/s41385-019-0205-x 31554901

[B6] Bai L. Gao J. Wei F. Zhao J. Wang D. Wei J. (2018). Therapeutic potential of ginsenosides as an adjuvant treatment for diabetes. Front. Pharmacol. 9, 423. 10.3389/fphar.2018.00423 29765322 PMC5938666

[B7] Bala V. Rajagopal S. Kumar D. P. Nalli A. D. Mahavadi S. Sanyal A. J. (2014). Release of GLP-1 and PYY in response to the activation of G protein-coupled bile acid receptor TGR5 is mediated by Epac/PLC-ε pathway and modulated by endogenous H2S. Front. Physiol. 5, 420. 10.3389/fphys.2014.00420 25404917 PMC4217307

[B8] Bellentani S. (2005). Immunomodulating and anti-apoptotic action of ursodeoxycholic acid: Where are we and where should we go? Eur. J. Gastroenterol. Hepatol. 17, 137–140. 10.1097/00042737-200502000-00001 15674088

[B9] Blin C. Passet V. Touchon M. Rocha E. Brisse S. (2017). Metabolic diversity of the emerging pathogenic lineages of *Klebsiella pneumoniae* . Environ. Microbiol. 19, 1881–1898. 10.1111/1462-2920.13689 28181409

[B10] Buwen J. P. Kammerer M. R. Beekley A. C. Tichansky D. S. (2015). Laparoscopic sleeve gastrectomy: The rightful gold standard weight loss surgery procedure. Surg. Obes. Relat. Dis. 11, 1383–1385. 10.1016/j.soard.2015.06.013 26278194

[B11] Cao H. Chen Z. X. Wang K. Ning M. M. Zou Q. A. Feng Y. (2016). Intestinally-targeted TGR5 agonists equipped with quaternary ammonium have an improved hypoglycemic effect and reduced gallbladder filling effect. Sci. Rep. 6, 28676. 10.1038/srep28676 27339735 PMC4919643

[B12] Carbajo-Pescador S. Porras D. García-Mediavilla M. V. Martínez-Flórez S. Juarez-Fernández M. Cuevas M. J. (2019). Beneficial effects of exercise on gut microbiota functionality and barrier integrity, and gut-liver crosstalk in an *in vivo* model of early obesity and non-alcoholic fatty liver disease. Dis. Model. Mech. 12, dmm039206. 10.1242/dmm.039206 30971408 PMC6550047

[B13] Cariou B. van Harmelen K. Duran-Sandoval D. van Dijk T. H. Grefhorst A. Abdelkarim M. (2006). The farnesoid X receptor modulates adiposity and peripheral insulin sensitivity in mice. J. Biol. Chem. 281, 11039–11049. 10.1074/jbc.M510258200 16446356

[B14] Castañeda T. R. Méndez M. Davison I. Elvert R. Schwahn U. Boldina G. (2021). The novel phosphate and bile acid sequestrant polymer SAR442357 delays disease progression in a rat model of diabetic nephropathy. J. Pharmacol. Exp. Ther. 376, 190–203. 10.1124/jpet.120.000285 33203659

[B15] Castellanos-Jankiewicz A. Guzmán-Quevedo O. Fénelon V. S. Zizzari P. Quarta C. Bellocchio L. (2021). Hypothalamic bile acid-TGR5 signaling protects from obesity. Cell. Metab. 33, 1483–1492.e10. e10. 10.1016/j.cmet.2021.04.009 33887197

[B16] Castro R. E. Ferreira D. M. Afonso M. B. Borralho P. M. Machado M. V. Cortez-Pinto H. (2013). miR-34a/SIRT1/p53 is suppressed by ursodeoxycholic acid in the rat liver and activated by disease severity in human non-alcoholic fatty liver disease. J. Hepatol. 58, 119–125. 10.1016/j.jhep.2012.08.008 22902550

[B17] Chambers A. P. Smith E. P. Begg D. P. Grayson B. E. Sisley S. Greer T. (2014). Regulation of gastric emptying rate and its role in nutrient-induced GLP-1 secretion in rats after vertical sleeve gastrectomy. Am. J. Physiol. Endocrinol. Metab. 306, E424–E432. 10.1152/ajpendo.00469.2013 24368666 PMC3923088

[B18] Chambers K. F. Day P. E. Aboufarrag H. T. Kroon P. A. (2019). Polyphenol effects on cholesterol metabolism via bile acid biosynthesis, CYP7A1: A review. Nutrients 11, 2588. 10.3390/nu11112588 PMC6893479 31661763

[B19] Chaudhari S. N. Harris D. A. Aliakbarian H. Luo J. N. Henke M. T. Subramaniam R. (2021). Bariatric surgery reveals a gut-restricted TGR5 agonist with anti-diabetic effects. Nat. Chem. Biol. 17, 20–29. 10.1038/s41589-020-0604-z 32747812 PMC7891870

[B20] Chen C. Hu B. Wu T. Zhang Y. Xu Y. Feng Y. (2016). Bile acid profiles in diabetic (db/db) mice and their wild type littermates. J. Pharm. Biomed. Anal. 131, 473–481. 10.1016/j.jpba.2016.09.023 27689719

[B21] Chen M. X. Wang S. Y. Kuo C. H. Tsai I. L. (2019). Metabolome analysis for investigating host-gut microbiota interactions. J. Formos. Med. Assoc. 118 (1), S10–S22. 10.1016/j.jfma.2018.09.007 30269936

[B22] Chiang J. Ferrell J. M. (2019). Bile acids as metabolic regulators and nutrient sensors. Annu. Rev. Nutr. 39, 175–200. 10.1146/annurev-nutr-082018-124344 31018107 PMC6996089

[B23] Chiang J. Y. (2013). Bile acid metabolism and signaling. Compr. Physiol. 3, 1191–1212. 10.1002/cphy.c120023 23897684 PMC4422175

[B24] Cipriani S. Mencarelli A. Chini M. G. Distrutti E. Renga B. Bifulco G. (2011). The bile acid receptor GPBAR-1 (TGR5) modulates integrity of intestinal barrier and immune response to experimental colitis. PLoS One 6, e25637. 10.1371/journal.pone.0025637 22046243 PMC3203117

[B25] Cipriani S. Mencarelli A. Palladino G. Fiorucci S. (2010). FXR activation reverses insulin resistance and lipid abnormalities and protects against liver steatosis in Zucker (fa/fa) obese rats. J. Lipid Res. 51, 771–784. 10.1194/jlr.M001602 19783811 PMC2842143

[B26] Comeglio P. Cellai I. Mello T. Filippi S. Maneschi E. Corcetto F. (2018). INT-767 prevents NASH and promotes visceral fat Brown adipogenesis and mitochondrial function. J. Endocrinol. 238, 107–127. 10.1530/JOE-17-0557 29945982

[B27] Cook J. R. Langlet F. Kido Y. Accili D. (2015). Pathogenesis of selective insulin resistance in isolated hepatocytes. J. Biol. Chem. 290, 13972–13980. 10.1074/jbc.M115.638197 25873396 PMC4447970

[B28] Costabile A. Buttarazzi I. Kolida S. Quercia S. Baldini J. Swann J. R. (2017). An *in vivo* assessment of the cholesterol-lowering efficacy of Lactobacillus plantarum ECGC 13110402 in normal to mildly hypercholesterolaemic adults. PLoS One 12, e0187964. 10.1371/journal.pone.0187964 29228000 PMC5724841

[B29] da Silva J. A. Jr Figueiredo L. S. Chaves J. O. Oliveira K. M. Carneiro E. M. Abreu P. A. (2021). Effects of tauroursodeoxycholic acid on glucose homeostasis: Potential binding of this bile acid with the insulin receptor. Life Sci. 285, 120020. 10.1016/j.lfs.2021.120020 34624320

[B30] Ðanić M. Stanimirov B. Pavlović N. Goločorbin-Kon S. Al-Salami H. Stankov K. (2018). Pharmacological applications of bile acids and their derivatives in the treatment of metabolic syndrome. Front. Pharmacol. 9, 1382. 10.3389/fphar.2018.01382 30559664 PMC6287190

[B31] de Aguiar Vallim T. Q. Tarling E. J. Edwards P. A. (2013). Pleiotropic roles of bile acids in metabolism. Cell. Metab. 17, 657–669. 10.1016/j.cmet.2013.03.013 23602448 PMC3654004

[B32] de Boer J. F. Schonewille M. Boesjes M. Wolters H. Bloks V. W. Bos T. (2017). Intestinal farnesoid X receptor controls transintestinal cholesterol excretion in mice. Gastroenterology 152, 1126–1138. e6. 10.1053/j.gastro.2016.12.037 28065787

[B33] Degirolamo C. Rainaldi S. Bovenga F. Murzilli S. Moschetta A. (2014). Microbiota modification with probiotics induces hepatic bile acid synthesis via downregulation of the Fxr-Fgf15 axis in mice. Cell. Rep. 7, 12–18. 10.1016/j.celrep.2014.02.032 24656817

[B34] Depommier C. Everard A. Druart C. Plovier H. Van Hul M. Vieira-Silva S. (2019). Supplementation with akkermansia muciniphila in overweight and obese human volunteers: A proof-of-concept exploratory study. Nat. Med. 25, 1096–1103. 10.1038/s41591-019-0495-2 31263284 PMC6699990

[B35] Ding L. Yang Q. Zhang E. Wang Y. Sun S. Yang Y. (2021a). Notoginsenoside Ft1 acts as a TGR5 agonist but FXR antagonist to alleviate high fat diet-induced obesity and insulin resistance in mice. Acta Pharm. Sin. B 11, 1541–1554. 10.1016/j.apsb.2021.03.038 34221867 PMC8245856

[B36] Ding L. Zhang E. Yang Q. Jin L. Sousa K. M. Dong B. (2021b). Vertical sleeve gastrectomy confers metabolic improvements by reducing intestinal bile acids and lipid absorption in mice. Proc. Natl. Acad. Sci. U. S. A. 118, e2019388118. 10.1073/pnas.2019388118 33526687 PMC8017941

[B37] Distrutti E. O'Reilly J. A. McDonald C. Cipriani S. Renga B. Lynch M. A. (2014). Modulation of intestinal microbiota by the probiotic VSL#3 resets brain gene expression and ameliorates the age-related deficit in LTP. PLoS One 9, e106503. 10.1371/journal.pone.0106503 25202975 PMC4159266

[B38] Duboc H. Rajca S. Rainteau D. Benarous D. Maubert M. A. Quervain E. (2013). Connecting dysbiosis, bile-acid dysmetabolism and gut inflammation in inflammatory bowel diseases. Gut 62, 531–539. 10.1136/gutjnl-2012-302578 22993202

[B39] Duboc H. Taché Y. Hofmann A. F. (2014). The bile acid TGR5 membrane receptor: From basic research to clinical application. Dig. Liver Dis. 46, 302–312. 10.1016/j.dld.2013.10.021 24411485 PMC5953190

[B40] Fang S. Suh J. M. Reilly S. M. Yu E. Osborn O. Lackey D. (2015). Intestinal FXR agonism promotes adipose tissue browning and reduces obesity and insulin resistance. Nat. Med. 21, 159–165. 10.1038/nm.3760 25559344 PMC4320010

[B41] Finn P. D. Rodriguez D. Kohler J. Jiang Z. Wan S. Blanco E. (2019). Intestinal TGR5 agonism improves hepatic steatosis and insulin sensitivity in Western diet-fed mice. Am. J. Physiol. Gastrointest. Liver Physiol. 316, G412–G424. 10.1152/ajpgi.00300.2018 30605011 PMC6459286

[B42] Fiorucci S. Cipriani S. Mencarelli A. Baldelli F. Bifulco G. Zampella A. (2011). Farnesoid X receptor agonist for the treatment of liver and metabolic disorders: Focus on 6-ethyl-CDCA. Mini Rev. Med. Chem. 11, 753–762. 10.2174/138955711796355258 21707532

[B43] Flachs P. Rossmeisl M. Kopecky J. (2014). The effect of n-3 fatty acids on glucose homeostasis and insulin sensitivity. Physiol. Res., S93–S118. 10.33549/physiolres.932715 24564669

[B44] Franzosa E. A. Sirota-Madi A. Avila-Pacheco J. Fornelos N. Haiser H. J. Reinker S. (2019). Gut microbiome structure and metabolic activity in inflammatory bowel disease. Nat. Microbiol. 4, 293–305. 10.1038/s41564-018-0306-4 30531976 PMC6342642

[B45] Gomez E. Pritchard C. Herbert T. P. (2002). cAMP-dependent protein kinase and Ca2+ influx through L-type voltage-gated calcium channels mediate Raf-independent activation of extracellular regulated kinase in response to glucagon-like peptide-1 in pancreatic beta-cells. J. Biol. Chem. 277, 48146–48151. 10.1074/jbc.M209165200 12364324

[B46] Gonzalez F. J. Jiang C. Patterson A. D. (2016). An intestinal microbiota-farnesoid X receptor Axis modulates metabolic disease. Gastroenterology 151, 845–859. 10.1053/j.gastro.2016.08.057 27639801 PMC5159222

[B47] Grander C. Adolph T. E. Wieser V. Lowe P. Wrzosek L. Gyongyosi B. (2018). Recovery of ethanol-induced Akkermansia muciniphila depletion ameliorates alcoholic liver disease. Gut 67, 891–901. 10.1136/gutjnl-2016-313432 28550049

[B48] Grandl G. Wolfrum C. (2018). Hemostasis, endothelial stress, inflammation, and the metabolic syndrome. Semin. Immunopathol. 40, 215–224. 10.1007/s00281-017-0666-5 29209827 PMC5809518

[B49] Guo C. Xie S. Chi Z. Zhang J. Liu Y. Zhang L. (2016). Bile acids control inflammation and metabolic disorder through inhibition of NLRP3 inflammasome. Immunity 45, 802–816. 10.1016/j.immuni.2016.09.008 27692610

[B50] Guo D. He L. Gao Y. Jin C. Lin H. Zhang L. (2021). Obeticholic acid derivative, T-2054 suppresses osteoarthritis via inhibiting NF-κB-Signaling pathway. Int. J. Mol. Sci. 22, 3807. 10.3390/ijms22083807 33916928 PMC8067620

[B51] Guo J. Y. Chen H. H. Lee W. J. Chen S. C. Lee S. D. Chen C. Y. (2022). Fibroblast growth factor 19 and fibroblast growth factor 21 regulation in obese diabetics, and non-alcoholic fatty liver disease after gastric bypass. Nutrients 14, 645. 10.3390/nu14030645 35277004 PMC8839096

[B52] Gupta J. Singh D. P. Verma P. C. Rahuja N. Srivastava R. Ahmad I. (2022). Pregnane-oximino-alkyl-amino-ether compound as a novel class of TGR5 receptor agonist exhibiting antidiabetic and anti-dyslipidemic activities. Pharmacology 107, 54–68. 10.1159/000519721 34814141

[B53] Haeusler R. A. Astiarraga B. Camastra S. Accili D. Ferrannini E. (2013). Human insulin resistance is associated with increased plasma levels of 12α-hydroxylated bile acids. Diabetes 62, 4184–4191. 10.2337/db13-0639 23884887 PMC3837033

[B54] Han S. Y. Song H. K. Cha J. J. Han J. Y. Kang Y. S. Cha D. R. (2021). Farnesoid X receptor (FXR) agonist ameliorates systemic insulin resistance, dysregulation of lipid metabolism, and alterations of various organs in a type 2 diabetic kidney animal model. Acta Diabetol. 58, 495–503. 10.1007/s00592-020-01652-z 33399988

[B55] Hao H. Cao L. Jiang C. Che Y. Zhang S. Takahashi S. (2017). Farnesoid X receptor regulation of the NLRP3 inflammasome underlies cholestasis-associated sepsis. Cell. Metab. 25, 856–867. e5. 10.1016/j.cmet.2017.03.007 28380377 PMC6624427

[B56] Harach T. Pols T. W. Nomura M. Maida A. Watanabe M. Auwerx J. (2012). TGR5 potentiates GLP-1 secretion in response to anionic exchange resins. Sci. Rep. 2, 430. 10.1038/srep00430 22666533 PMC3362799

[B57] Hartmann P. Duan Y. Miyamoto Y. Demir M. Lang S. Hasa E. (2022). Colesevelam ameliorates non-alcoholic steatohepatitis and obesity in mice. Hepatol. Int. 16, 359–370. 10.1007/s12072-022-10296-w 35075592 PMC9013343

[B58] Hernández E. Á. Kahl S. Seelig A. Begovatz P. Irmler M. Kupriyanova Y. (2017). Acute dietary fat intake initiates alterations in energy metabolism and insulin resistance. J. Clin. Invest. 127, 695–708. 10.1172/JCI89444 28112681 PMC5272194

[B59] Higuchi S. Ahmad T. R. Argueta D. A. Perez P. A. Zhao C. Schwartz G. J. (2020). Bile acid composition regulates GPR119-dependent intestinal lipid sensing and food intake regulation in mice. Gut 69, 1620–1628. 10.1136/gutjnl-2019-319693 32111630 PMC7423635

[B60] Hira T. Pinyo J. Hara H. (2020). What is GLP-1 really doing in obesity? Trends Endocrinol. Metab. 31, 71–80. 10.1016/j.tem.2019.09.003 31636017

[B61] Holland W. L. Adams A. C. Brozinick J. T. Bui H. H. Miyauchi Y. Kusminski C. M. (2013). An FGF21-adiponectin-ceramide axis controls energy expenditure and insulin action in mice. Cell. Metab. 17, 790–797. 10.1016/j.cmet.2013.03.019 23663742 PMC3667496

[B62] Hosseinkhani F. Heinken A. Thiele I. Lindenburg P. W. Harms A. C. Hankemeier T. (2021). The contribution of gut bacterial metabolites in the human immune signaling pathway of non-communicable diseases. Gut Microbes 13, 1–22. 10.1080/19490976.2021.1882927 PMC7899087 33590776

[B63] Hu J. Wang C. Huang X. Yi S. Pan S. Zhang Y. (2021). Gut microbiota-mediated secondary bile acids regulate dendritic cells to attenuate autoimmune uveitis through TGR5 signaling. Cell. Rep. 36, 109726. 10.1016/j.celrep.2021.109726 34551302

[B64] Hu J. Zhang Y. Yi S. Wang C. Huang X. Pan S. (2022). Lithocholic acid inhibits dendritic cell activation by reducing intracellular glutathione via TGR5 signaling. Int. J. Biol. Sci. 18, 4545–4559. 10.7150/ijbs.71287 35864954 PMC9295063

[B65] Huang S. Ma S. Ning M. Yang W. Ye Y. Zhang L. (2019). TGR5 agonist ameliorates insulin resistance in the skeletal muscles and improves glucose homeostasis in diabetic mice. Metabolism. 99, 45–56. 10.1016/j.metabol.2019.07.003 31295453

[B66] Inagaki T. Choi M. Moschetta A. Peng L. Cummins C. L. McDonald J. G. (2005). Fibroblast growth factor 15 functions as an enterohepatic signal to regulate bile acid homeostasis. Cell. Metab. 2, 217–225. 10.1016/j.cmet.2005.09.001 16213224

[B67] International Diabetes Federation (2021). IDF diabetes atlas. 10th Edition. Bookshelf ID, NBK581934. 35914061

[B68] Islam K. B. Fukiya S. Hagio M. Fujii N. Ishizuka S. Ooka T. (2011). Bile acid is a host factor that regulates the composition of the cecal microbiota in rats. Gastroenterology 141, 1773–1781. 10.1053/j.gastro.2011.07.046 21839040

[B69] Jena P. K. Sheng L. Li Y. Wan Y. Y. (2020). Probiotics VSL#3 are effective in reversing non-alcoholic steatohepatitis in a mouse model. Hepatobiliary Surg. Nutr. 9, 170–182. 10.21037/hbsn.2019.09.07 32355675 PMC7188546

[B70] Jia H. Y. Yang C. Q. (2019). The role of enterohepatic circulation of bile acids and intestinal microbiota in the pathogenesis and treatment of cholestatic liver disease. J. Clin. Hepatol. 35, 270–274. 10.3969/j.issn.1001-5256.2019.02.007

[B71] Jia W. Wei M. Rajani C. Zheng X. (2021). Targeting the alternative bile acid synthetic pathway for metabolic diseases. Protein Cell. 12, 411–425. 10.1007/s13238-020-00804-9 33252713 PMC8106556

[B72] Jiang C. Xie C. Lv Y. Li J. Krausz K. W. Shi J. (2015). Intestine-selective farnesoid X receptor inhibition improves obesity-related metabolic dysfunction. Nat. Commun. 6, 10166. 10.1038/ncomms10166 26670557 PMC4682112

[B73] Jiang L. S. Li W. Zhuang T. X. Yu J. J. Sun S. Ju Z. C. (2021). Ginsenoside Ro ameliorates high-fat diet-induced obesity and insulin resistance in mice via activation of the G protein-coupled bile acid receptor 5 pathway. J. Pharmacol. Exp. Ther. 377, 441–451. 10.1124/jpet.120.000435 33820830

[B74] Kakiyama G. Pandak W. M. Gillevet P. M. Hylemon P. B. Heuman D. M. Daita K. (2013). Modulation of the fecal bile acid profile by gut microbiota in cirrhosis. J. Hepatol. 58, 949–955. 10.1016/j.jhep.2013.01.003 23333527 PMC3936319

[B75] Kaur A. Patankar J. V. de Haan W. Ruddle P. Wijesekara N. Groen A. K. (2015). Loss of Cyp8b1 improves glucose homeostasis by increasing GLP-1. Diabetes 64, 1168–1179. 10.2337/db14-0716 25338812

[B76] Kir S. Beddow S. A. Samuel V. T. Miller P. Previs S. F. Suino-Powell K. (2011). FGF19 as a postprandial, insulin-independent activator of hepatic protein and glycogen synthesis. Science 331, 1621–1624. 10.1126/science.1198363 21436455 PMC3076083

[B77] Kökten T. Hansmannel F. Ndiaye N. C. Heba A. C. Quilliot D. Dreumont N. (2021). Calorie restriction as a new treatment of inflammatory diseases. Adv. Nutr. 12, 1558–1570. 10.1093/advances/nmaa179 33554240 PMC8321869

[B78] Kuhre R. E. Holst J. J. Kappe C. (2016). The regulation of function, growth and survival of GLP-1-producing L-cells. Clin. Sci. 130, 79–91. 10.1042/CS20150154 26637406

[B79] Kumar D. P. Asgharpour A. Mirshahi F. Park S. H. Liu S. Imai Y. (2016). Activation of transmembrane bile acid receptor TGR5 modulates pancreatic islet α cells to promote glucose homeostasis. J. Biol. Chem. 291, 6626–6640. 10.1074/jbc.M115.699504 26757816 PMC4807250

[B80] Kuro-O. M. (2019). The Klotho proteins in health and disease. Nat. Rev. Nephrol. 15, 27–44. 10.1038/s41581-018-0078-3 30455427

[B81] Lan T. Morgan D. A. Rahmouni K. Sonoda J. Fu X. Burgess S. C. (2017). FGF19, FGF21, and an FGFR1/β-klotho-activating antibody act on the nervous system to regulate body weight and glycemia. Cell. Metab. 26, 709–718. e3. 10.1016/j.cmet.2017.09.005 28988823 PMC5679468

[B82] Lasalle M. Hoguet V. Hennuyer N. Leroux F. Piveteau C. Belloy L. (2017). Topical intestinal aminoimidazole agonists of G-protein-coupled bile acid receptor 1 promote glucagon like peptide-1 secretion and improve glucose tolerance. J. Med. Chem. 60, 4185–4211. 10.1021/acs.jmedchem.6b01873 28414465

[B83] Legry V. Francque S. Haas J. T. Verrijken A. Caron S. Chávez-Talavera O. (2017). Bile acid alterations are associated with insulin resistance, but not with NASH, in obese subjects. J. Clin. Endocrinol. Metab. 102, 3783–3794. 10.1210/jc.2017-01397 28938455

[B84] Li B. Yang N. Li C. Li C. Gao K. Xie X. (2018). INT-777, a bile acid receptor agonist, extenuates pancreatic acinar cells necrosis in a mouse model of acute pancreatitis. Biochem. Biophys. Res. Commun. 503, 38–44. 10.1016/j.bbrc.2018.05.120 29859191

[B85] Li J. Li T. (2017). Bile acid receptors link nutrient sensing to metabolic regulation. Liver Res. 1, 17–25. 10.1016/j.livres.2017.04.001 29098111 PMC5662125

[B86] Li M. Wang S. Li Y. Zhao M. Kuang J. Liang D. (2022). Gut microbiota-bile acid crosstalk contributes to the rebound weight gain after calorie restriction in mice. Nat. Commun. 13, 2060. 10.1038/s41467-022-29589-7 35440584 PMC9018700

[B87] Li T. Chiang J. Y. (2014). Bile acid signaling in metabolic disease and drug therapy. Pharmacol. Rev. 66, 948–983. 10.1124/pr.113.008201 25073467 PMC4180336

[B88] Liang C. Zhou X. H. Gong P. M. Niu H. Y. Lyu L. Z. Wu Y. F. (2021). Lactiplantibacillus plantarum H-87 prevents high-fat diet-induced obesity by regulating bile acid metabolism in C57BL/6J mice. Food Funct. 12, 4315–4324. 10.1039/d1fo00260k 34031676

[B89] Liu D. Huang Y. Huang C. Yang S. Wei X. Zhang P. (2022). Calorie restriction with or without time-restricted eating in weight loss. N. Engl. J. Med. 386, 1495–1504. 10.1056/NEJMoa2114833 35443107

[B90] Liu S. Marcelin G. Blouet C. Jeong J. H. Jo Y. H. Schwartz G. J. (2018). A gut-brain axis regulating glucose metabolism mediated by bile acids and competitive fibroblast growth factor actions at the hypothalamus. Mol. Metab. 8, 37–50. 10.1016/j.molmet.2017.12.003 29290621 PMC5985052

[B91] Lloyd-Price J. Arze C. Ananthakrishnan A. N. Schirmer M. Avila-Pacheco J. Poon T. W. (2019). Multi-omics of the gut microbial ecosystem in inflammatory bowel diseases. Nature 569, 655–662. 10.1038/s41586-019-1237-9 31142855 PMC6650278

[B92] Long S. L. Gahan C. Joyce S. A. (2017). Interactions between gut bacteria and bile in health and disease. Mol. Asp. Med. 56, 54–65. 10.1016/j.mam.2017.06.002 28602676

[B93] Lukivskaya O. Patsenker E. Buko V. U. (2007). Protective effect of ursodeoxycholic acid on liver mitochondrial function in rats with alloxan-induced diabetes: Link with oxidative stress. Life Sci. 80, 2397–2402. 10.1016/j.lfs.2007.02.042 17512017

[B94] Lye H. S. Rusul G. Liong M. T. (2010). Removal of cholesterol by lactobacilli via incorporation and conversion to coprostanol. J. Dairy Sci. 93, 1383–1392. 10.3168/jds.2009-2574 20338415

[B95] Marlatt K. L. Redman L. M. Burton J. H. Martin C. K. Ravussin E. (2017). Persistence of weight loss and acquired behaviors 2 y after stopping a 2-y calorie restriction intervention. Am. J. Clin. Nutr. 105, 928–935. 10.3945/ajcn.116.146837 28275127 PMC5366052

[B96] Martoni C. J. Labbé A. Ganopolsky J. G. Prakash S. Jones M. L. (2015). Changes in bile acids, FGF-19 and sterol absorption in response to bile salt hydrolase active L reuteri NCIMB 30242. Gut Microbes 6, 57–65. 10.1080/19490976.2015.1005474 25612224 PMC4615650

[B97] Massey W. Brown J. M. (2021). The gut microbial endocrine organ in type 2 diabetes. Endocrinology 162, 235. 10.1210/endocr/bqaa235 PMC7806240 33373432

[B98] Mathur B. Shajahan A. Arif W. Chen Q. Hand N. J. Abramowitz L. K. (2021). Nuclear receptors FXR and SHP regulate protein N-glycan modifications in the liver. Sci. Adv. 7, eabf4865. 10.1126/sciadv.abf4865 33883138 PMC8059921

[B99] Meier P. J. Stieger B. (2002). Bile salt transporters. Annu. Rev. Physiol. 64, 635–661. 10.1146/annurev.physiol.64.082201.100300 11826283

[B100] Mingrone G. Panunzi S. De Gaetano A. Guidone C. Iaconelli A. Nanni G. (2015). Bariatric-metabolic surgery versus conventional medical treatment in obese patients with type 2 diabetes: 5 year follow-up of an open-label, single-centre, randomised controlled trial. Lancet 386, 964–973. 10.1016/S0140-6736(15)00075-6 26369473

[B101] Miyata M. Takamatsu Y. Kuribayashi H. Yamazoe Y. (2009). Administration of ampicillin elevates hepatic primary bile acid synthesis through suppression of ileal fibroblast growth factor 15 expression. J. Pharmacol. Exp. Ther. 331, 1079–1085. 10.1124/jpet.109.160093 19767447

[B102] Mooranian A. Negrulj R. Al-Salami H. Morahan G. Jamieson E. (2016). Designing anti-diabetic β-cells microcapsules using polystyrenic sulfonate, polyallylamine, and a tertiary bile acid: Morphology, bioenergetics, and cytokine analysis. Biotechnol. Prog. 32, 501–509. 10.1002/btpr.2223 26748789

[B103] Mooranian A. Zamani N. Kovacevic B. Ionescu C. M. Luna G. Mikov M. (2022). Pharmacological effects of secondary bile acid microparticles in diabetic murine model. Curr. Diabetes Rev. 18, e062620183199. 10.2174/1573399816666200626213735 32589561

[B104] Morimoto K. Watanabe M. Sugizaki T. Irie J. Itoh H. (2016). Intestinal bile acid composition modulates prohormone convertase 1/3 (PC1/3) expression and consequent GLP-1 production in male mice. Endocrinology 157, 1071–1081. 10.1210/en.2015-1551 26789236

[B105] Mudaliar S. Henry R. R. Sanyal A. J. Morrow L. Marschall H. U. Kipnes M. (2013). Efficacy and safety of the farnesoid X receptor agonist obeticholic acid in patients with type 2 diabetes and nonalcoholic fatty liver disease. Gastroenterology 145, 574–582. 10.1053/j.gastro.2013.05.042 23727264

[B106] Ni W. Glenn D. J. Gardner D. G. (2016). Tie-2Cre mediated deletion of the vitamin D receptor gene leads to improved skeletal muscle insulin sensitivity and glucose tolerance. J. Steroid Biochem. Mol. Biol. 164, 281–286. 10.1016/j.jsbmb.2015.09.017 26369613 PMC4788578

[B107] Ni Y. Zhao Y. Ma L. Wang Z. Ni L. Hu L. (2021). Pharmacological activation of REV-ERBα improves nonalcoholic steatohepatitis by regulating intestinal permeability. Metabolism. 114, 154409. 10.1016/j.metabol.2020.154409 33096076

[B108] Nie B. Park H. M. Kazantzis M. Lin M. Henkin A. Ng S. (2012). Specific bile acids inhibit hepatic fatty acid uptake in mice. Hepatology 56, 1300–1310. 10.1002/hep.25797 22531947 PMC3445775

[B109] Noel O. F. Still C. D. Argyropoulos G. Edwards M. Gerhard G. S. (2016). Bile acids, FXR, and metabolic effects of bariatric surgery. J. Obes. 2016, 4390254. 10.1155/2016/4390254 27006824 PMC4783581

[B110] Odermatt A. Da Cunha T. Penno C. A. Chandsawangbhuwana C. Reichert C. Wolf A. (2011). Hepatic reduction of the secondary bile acid 7-oxolithocholic acid is mediated by 11β-hydroxysteroid dehydrogenase 1. Biochem. J. 436, 621–629. 10.1042/BJ20110022 21453287

[B111] Oh A. R. Bae J. S. Lee J. Shin E. Oh B. C. Park S. C. (2016). Ursodeoxycholic acid decreases age-related adiposity and inflammation in mice. BMB Rep. 49, 105–110. 10.5483/bmbrep.2016.49.2.173 26350747 PMC4915113

[B112] Oh J. Riek A. E. Darwech I. Funai K. Shao J. Chin K. (2015). Deletion of macrophage Vitamin D receptor promotes insulin resistance and monocyte cholesterol transport to accelerate atherosclerosis in mice. Cell. Rep. 10, 1872–1886. 10.1016/j.celrep.2015.02.043 25801026 PMC4495012

[B113] Öner Ö. Aslim B. Aydaş S. B. (2014). Mechanisms of cholesterol-lowering effects of lactobacilli and bifidobacteria strains as potential probiotics with their bsh gene analysis. J. Mol. Microbiol. Biotechnol. 24, 12–18. 10.1159/000354316 24158048

[B114] Ordonez R. Carbajo-Pescador S. Mauriz J. L. Gonzalez-Gallego J. (2015). Understanding nutritional interventions and physical exercise in non-alcoholic fatty liver disease. Curr. Mol. Med. 15, 3–26. 10.2174/1566524015666150114110551 25601465

[B115] Pandak W. M. Kakiyama G. (2019). The acidic pathway of bile acid synthesis: Not just an alternative pathway. Liver Res. 3, 88–98. 10.1016/j.livres.2019.05.001 32015930 PMC6996149

[B116] Pathak P. Chiang J. (2019). Sterol 12α-hydroxylase aggravates dyslipidemia by activating the ceramide/mTORC1/SREBP-1C pathway via FGF21 and FGF15. Gene Expr. 19, 161–173. 10.3727/105221619X15529371970455 30890204 PMC6827038

[B117] Pathak P. Xie C. Nichols R. G. Ferrell J. M. Boehme S. Krausz K. W. (2018). Intestine farnesoid X receptor agonist and the gut microbiota activate G-protein bile acid receptor-1 signaling to improve metabolism. Hepatology 68, 1574–1588. 10.1002/hep.29857 29486523 PMC6111007

[B118] Perino A. Schoonjans K. (2022). Metabolic messengers: Bile acids. Nat. Metab. 4, 416–423. 10.1038/s42255-022-00559-z 35338368

[B119] Potthoff M. J. Boney-Montoya J. Choi M. He T. Sunny N. E. Satapati S. (2011). FGF15/19 regulates hepatic glucose metabolism by inhibiting the CREB-PGC-1α pathway. Cell. Metab. 13, 729–738. 10.1016/j.cmet.2011.03.019 21641554 PMC3131185

[B120] Potthoff M. J. Potts A. He T. Duarte J. A. Taussig R. Mangelsdorf D. J. (2013). Colesevelam suppresses hepatic glycogenolysis by TGR5-mediated induction of GLP-1 action in DIO mice. Am. J. Physiol. Gastrointest. Liver Physiol. 304, G371–G380. 10.1152/ajpgi.00400.2012 23257920 PMC3566618

[B121] Prawitt J. Abdelkarim M. Stroeve J. H. Popescu I. Duez H. Velagapudi V. R. (2011). Farnesoid X receptor deficiency improves glucose homeostasis in mouse models of obesity. Diabetes 60, 1861–1871. 10.2337/db11-0030 21593203 PMC3121443

[B122] Pushpass R. G. Alzoufairi S. Jackson K. G. Lovegrove J. A. (2021). Circulating bile acids as a link between the gut microbiota and cardiovascular health: Impact of prebiotics, probiotics and polyphenol-rich foods. Nutr. Res. Rev., 1–20. 10.1017/S0954422421000081 33926590

[B123] Qiu Y. Shen L. Fu L. Yang J. Cui C. Li T. (2020). The glucose-lowering effects of α-glucosidase inhibitor require a bile acid signal in mice. Diabetologia 63, 1002–1016. 10.1007/s00125-020-05095-7 32034442 PMC7145781

[B124] Quintero P. Pizarro M. Solís N. Arab J. P. Padilla O. Riquelme A. (2014). Bile acid supplementation improves established liver steatosis in obese mice independently of glucagon-like peptide-1 secretion. J. Physiol. Biochem. 70, 667–674. 10.1007/s13105-014-0336-1 24816727

[B125] Rao A. Kosters A. Mells J. E. Zhang W. Setchell K. D. Amanso A. M. (2016). Inhibition of ileal bile acid uptake protects against nonalcoholic fatty liver disease in high-fat diet-fed mice. Sci. Transl. Med. 8, 357ra122. 357ra122. 10.1126/scitranslmed.aaf4823 PMC5056562 27655848

[B126] Rao Y. Kuang Z. Li C. Guo S. Xu Y. Zhao D. (2021). Gut Akkermansia muciniphila ameliorates metabolic dysfunction-associated fatty liver disease by regulating the metabolism of L-aspartate via gut-liver axis. Gut Microbes 13, 1–19. 10.1080/19490976.2021.1927633 PMC8158032 34030573

[B127] Renga B. Mencarelli A. Cipriani S. D'Amore C. Carino A. Bruno A. (2013). The bile acid sensor FXR is required for immune-regulatory activities of TLR-9 in intestinal inflammation. PLoS One 8, e54472. 10.1371/journal.pone.0054472 23372731 PMC3555871

[B128] Rizzolo D. Kong B. Taylor R. E. Brinker A. Goedken M. Buckley B. (2021). Bile acid homeostasis in female mice deficient in Cyp7a1 and Cyp27a1. Acta Pharm. Sin. B 11, 3847–3856. 10.1016/j.apsb.2021.05.023 35024311 PMC8727763

[B129] Roden M. Shulman G. I. (2019). The integrative biology of type 2 diabetes. Nature 576, 51–60. 10.1038/s41586-019-1797-8 31802013

[B130] Sasaki H. Masuno H. Kawasaki H. Yoshihara A. Numoto N. Ito N. (2021). Lithocholic acid derivatives as potent vitamin D receptor agonists. J. Med. Chem. 64, 516–526. 10.1021/acs.jmedchem.0c01420 33369416

[B131] Sayin S. I. Wahlström A. Felin J. Jäntti S. Marschall H. U. Bamberg K. (2013). Gut microbiota regulates bile acid metabolism by reducing the levels of tauro-beta-muricholic acid, a naturally occurring FXR antagonist. Cell. Metab. 17, 225–235. 10.1016/j.cmet.2013.01.003 23395169

[B132] Schoeler M. Caesar R. (2019). Dietary lipids, gut microbiota and lipid metabolism. Rev. Endocr. Metab. Disord. 20, 461–472. 10.1007/s11154-019-09512-0 31707624 PMC6938793

[B133] Shapiro H. Kolodziejczyk A. A. Halstuch D. Elinav E. (2018). Bile acids in glucose metabolism in health and disease. J. Exp. Med. 215, 383–396. 10.1084/jem.20171965 29339445 PMC5789421

[B134] Sheng L. Jena P. K. Liu H. X. Hu Y. Nagar N. Bronner D. N. (2018). Obesity treatment by epigallocatechin-3-gallate-regulated bile acid signaling and its enriched Akkermansia muciniphila. FASEB J. 32, 6371–6384. 10.1096/fj.201800370R PMC6219838 29882708

[B135] Somm E. Jornayvaz F. R. (2018). Fibroblast growth factor 15/19: From basic functions to therapeutic perspectives. Endocr. Rev. 39, 960–989. 10.1210/er.2018-00134 30124818

[B136] Sonne D. P. van Nierop F. S. Kulik W. Soeters M. R. Vilsbøll T. Knop F. K. (2016). Postprandial plasma concentrations of individual bile acids and FGF-19 in patients with type 2 diabetes. J. Clin. Endocrinol. Metab. 101, 3002–3009. 10.1210/jc.2016-1607 27270475

[B137] Spinelli V. Lalloyer F. Baud G. Osto E. Kouach M. Daoudi M. (2016). Influence of roux-en-Y gastric bypass on plasma bile acid profiles: A comparative study between rats, pigs and humans. Int. J. Obes. (Lond) 40, 1260–1267. 10.1038/ijo.2016.46 27089995

[B138] Stepanov V. Stankov K. Mikov M. (2013). The bile acid membrane receptor TGR5: A novel pharmacological target in metabolic, inflammatory and neoplastic disorders. J. Recept. Signal Transduct. Res. 33, 213–223. 10.3109/10799893.2013.802805 23782454

[B139] Sun L. Xie C. Wang G. Wu Y. Wu Q. Wang X. (2018). Gut microbiota and intestinal FXR mediate the clinical benefits of metformin. Nat. Med. 24, 1919–1929. 10.1038/s41591-018-0222-4 30397356 PMC6479226

[B140] Ticho A. L. Malhotra P. Dudeja P. K. Gill R. K. Alrefai W. A. (2019). Intestinal absorption of bile acids in health and disease. Compr. Physiol. 10, 21–56. 10.1002/cphy.c190007 31853951 PMC7171925

[B141] Tomaro-Duchesneau C. LeValley S. L. Roeth D. Sun L. Horrigan F. T. Kalkum M. (2020). Discovery of a bacterial peptide as a modulator of GLP-1 and metabolic disease. Sci. Rep. 10 (1), 4922. 10.1038/s41598-020-61112-0 32188864 PMC7080827

[B142] Trabelsi M. S. Daoudi M. Prawitt J. Ducastel S. Touche V. Sayin S. I. (2015). Farnesoid X receptor inhibits glucagon-like peptide-1 production by enteroendocrine L cells. Nat. Commun. 6, 7629. 10.1038/ncomms8629 26134028 PMC4579574

[B143] Trabelsi M. S. Lestavel S. Staels B. Collet X. (2017). Intestinal bile acid receptors are key regulators of glucose homeostasis. Proc. Nutr. Soc. 76, 192–202. 10.1017/S0029665116002834 27846919

[B144] Velazquez-Villegas L. A. Perino A. Lemos V. Zietak M. Nomura M. Pols T. (2018). TGR5 signalling promotes mitochondrial fission and beige remodelling of white adipose tissue. Nat. Commun. 9, 245. 10.1038/s41467-017-02068-0 29339725 PMC5770450

[B145] Wahlström A. Sayin S. I. Marschall H. U. Bäckhed F. (2016). Intestinal crosstalk between bile acids and microbiota and its impact on host metabolism. Cell. Metab. 24, 41–50. 10.1016/j.cmet.2016.05.005 27320064

[B146] Wang X. H. Xu F. Cheng M. Wang X. Zhang D. M. Zhao L. H. (2020). Fasting serum total bile acid levels are associated with insulin sensitivity, islet β-cell function and glucagon levels in response to glucose challenge in patients with type 2 diabetes. Endocr. J. 67, 1107–1117. 10.1507/endocrj.EJ20-0201 32684527

[B147] Wei M. Huang F. Zhao L. Zhang Y. Yang W. Wang S. (2020). A dysregulated bile acid-gut microbiota axis contributes to obesity susceptibility. EBioMedicine 55, 102766. 10.1016/j.ebiom.2020.102766 32408110 PMC7225614

[B148] Weichhart T. Hengstschläger M. Linke M. (2015). Regulation of innate immune cell function by mTOR. Nat. Rev. Immunol. 15, 599–614. 10.1038/nri3901 26403194 PMC6095456

[B149] Wishart D. S. (2019). Metabolomics for investigating physiological and pathophysiological processes. Physiol. Rev. 99, 1819–1875. 10.1152/physrev.00035.2018 31434538

[B150] Wu H. Esteve E. Tremaroli V. Khan M. T. Caesar R. Mannerås-Holm L. (2017). Metformin alters the gut microbiome of individuals with treatment-naive type 2 diabetes, contributing to the therapeutic effects of the drug. Nat. Med. 23, 850–858. 10.1038/nm.4345 28530702

[B151] Wu L. Mo W. Feng J. Li J. Yu Q. Li S. (2020). Astaxanthin attenuates hepatic damage and mitochondrial dysfunction in non-alcoholic fatty liver disease by up-regulating the FGF21/PGC-1α pathway. Br. J. Pharmacol. 177, 3760–3777. 10.1111/bph.15099 32446270 PMC7393201

[B152] Wu X. Lv Y. G. Du Y. F. Hu M. Reed M. N. Long Y. (2019). Inhibitory effect of INT-777 on lipopolysaccharide-induced cognitive impairment, neuroinflammation, apoptosis, and synaptic dysfunction in mice. Prog. Neuropsychopharmacol. Biol. Psychiatry 88, 360–374. 10.1016/j.pnpbp.2018.08.016 30144494

[B153] Xiao H. Sun X. Liu R. Chen Z. Lin Z. Yang Y. (2020). Gentiopicroside activates the bile acid receptor Gpbar1 (TGR5) to repress NF-kappaB pathway and ameliorate diabetic nephropathy. Pharmacol. Res. 151, 104559. 10.1016/j.phrs.2019.104559 31759089

[B154] Xie C. Jiang C. Shi J. Gao X. Sun D. Sun L. (2017). An intestinal farnesoid X receptor-ceramide signaling Axis modulates hepatic gluconeogenesis in mice. Diabetes 66, 613–626. 10.2337/db16-0663 28223344 PMC5319721

[B155] Younge N. Yang Q. Seed P. C. (2017). Enteral high fat-polyunsaturated fatty acid blend alters the pathogen composition of the intestinal microbiome in premature infants with an enterostomy. J. Pediatr. 181, 93–101. e6. 10.1016/j.jpeds.2016.10.053 27856001 PMC5274578

[B156] Zangerolamo L. Vettorazzi J. F. Solon C. Bronczek G. A. Engel D. F. Kurauti M. A. (2021). The bile acid TUDCA improves glucose metabolism in streptozotocin-induced Alzheimer's disease mice model. Mol. Cell. Endocrinol. 521, 111116. 10.1016/j.mce.2020.111116 33321116

[B157] Zhai H. Li Z. Peng M. Huang Z. Qin T. Chen L. (2018). Takeda G protein-coupled receptor 5-mechanistic target of rapamycin complex 1 signaling contributes to the increment of glucagon-like peptide-1 production after roux-en-Y gastric bypass. EBioMedicine 32, 201–214. 10.1016/j.ebiom.2018.05.026 29859856 PMC6020750

[B158] Zhang L. Wu W. Lee Y. K. Xie J. Zhang H. (2018). Spatial heterogeneity and Co-occurrence of mucosal and luminal microbiome across swine intestinal tract. Front. Microbiol. 9, 48. 10.3389/fmicb.2018.00048 29472900 PMC5810300

[B159] Zhang T. Li Q. Cheng L. Buch H. Zhang F. (2019). Akkermansia muciniphila is a promising probiotic. Microb. Biotechnol. 12, 1109–1125. 10.1111/1751-7915.13410 31006995 PMC6801136

[B160] Zhao G. Dharmadhikari G. Maedler K. Meyer-Hermann M. (2014). Possible role of interleukin-1β in type 2 diabetes onset and implications for anti-inflammatory therapy strategies. PLoS Comput. Biol. 10, e1003798. 10.1371/journal.pcbi.1003798 25167060 PMC4148195

[B161] Zhao S. Liu W. Wang J. Shi J. Sun Y. Wang W. (2017). Akkermansia muciniphila improves metabolic profiles by reducing inflammation in chow diet-fed mice. J. Mol. Endocrinol. 58, 1–14. 10.1530/JME-16-0054 27821438

[B162] Zheng X. Chen T. Jiang R. Zhao A. Wu Q. Kuang J. (2021). Hyocholic acid species improve glucose homeostasis through a distinct TGR5 and FXR signaling mechanism. Cell. Metab. 33, 791–803.e7. 10.1016/j.cmet.2020.11.017 33338411

[B163] Zhuang P. Li H. Jia W. Shou Q. Zhu Y. Mao L. (2021). Eicosapentaenoic and docosahexaenoic acids attenuate hyperglycemia through the microbiome-gut-organs axis in db/db mice. Microbiome 9, 185. 10.1186/s40168-021-01126-6 34507608 PMC8434703

