# Supplementary figures and images for: Bile acids-gut microbiota crosstalk contributes to the improvement of type 2 diabetes mellitus
Source: Front Pharmacol. 2022 Oct 25;13:1027212. doi: 10.3389/fphar.2022.1027212 (PMC9640995; doi:10.3389/fphar.2022.1027212)

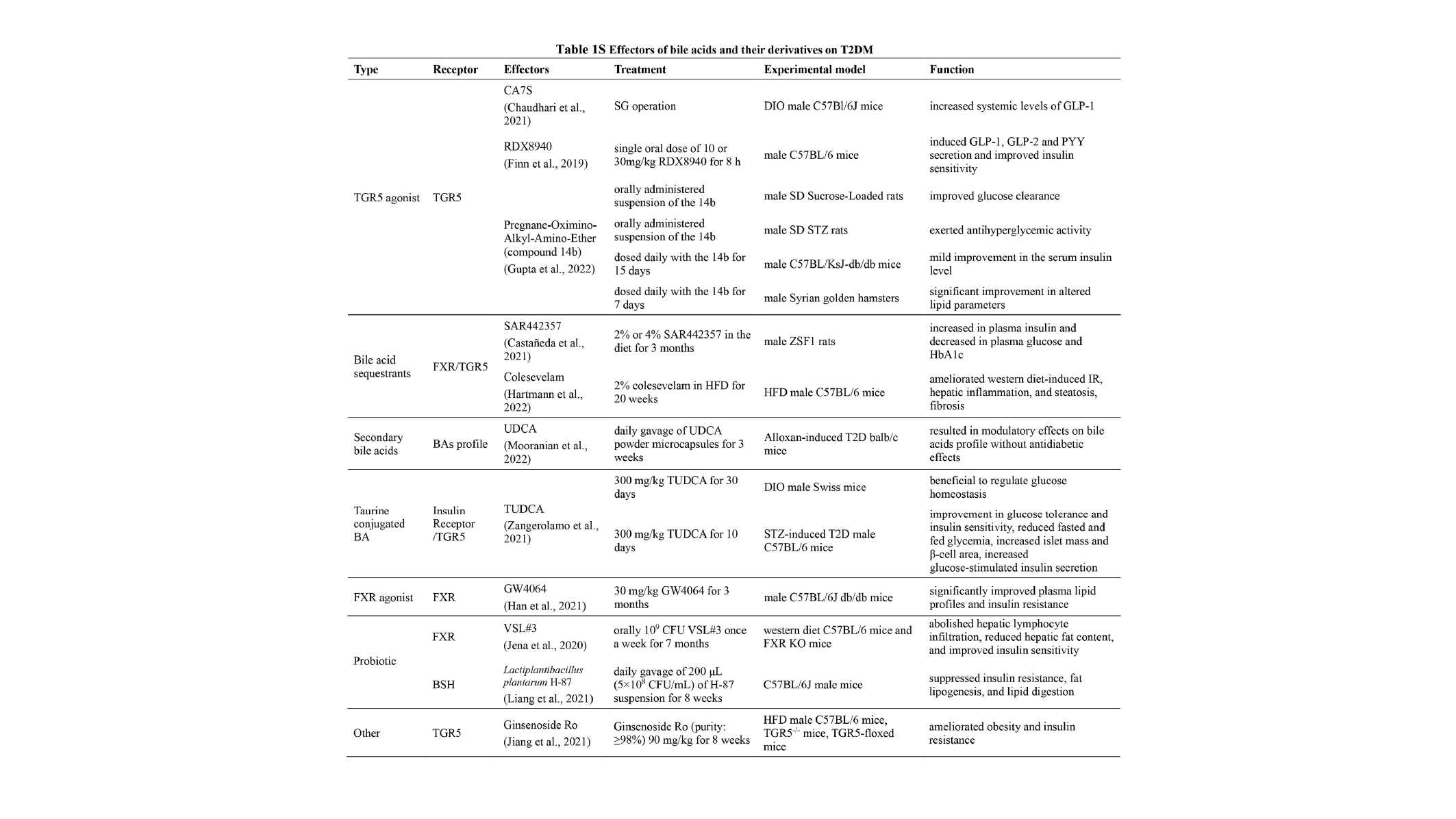

Supplement: Supplementary file 1 [file Image1.TIF]
